# Metabolic network reconstruction and genome-scale model of butanol-producing strain *Clostridium beijerinckii *NCIMB 8052

**DOI:** 10.1186/1752-0509-5-130

**Published:** 2011-08-16

**Authors:** Caroline B Milne, James A Eddy, Ravali Raju, Soroush Ardekani, Pan-Jun Kim, Ryan S Senger, Yong-Su Jin, Hans P Blaschek, Nathan D Price

**Affiliations:** 1 Department of Chemical and Biomolecular Engineering, University of Illinois, Urbana, IL, USA; 2 Institute for Genomic Biology, University of Illinois, Urbana, IL, USA; 3 Department of Bioengineering, University of Illinois, Urbana, IL, USA; 4 Center for Advanced BioEnergy Research, University of Illinois, Urbana, IL, USA; 5 Department of Biological Systems Engineering, Virginia Tech, Blacksburg, VA, USA; 6 Department of Food Science and Human Nutrition, University of Illinois, Urbana, IL, USA; 7 Department of Chemical Engineering and Material Science, University of Minnesota, MN, USA; 8 Institute for Systems Biology, 401 Terry Avenue N, Seattle, WA 98109, USA

## Abstract

**Background:**

Solventogenic clostridia offer a sustainable alternative to petroleum-based production of butanol--an important chemical feedstock and potential fuel additive or replacement. *C. beijerinckii *is an attractive microorganism for strain design to improve butanol production because it (i) naturally produces the highest recorded butanol concentrations as a byproduct of fermentation; and (ii) can co-ferment pentose and hexose sugars (the primary products from lignocellulosic hydrolysis). Interrogating *C. beijerinckii *metabolism from a systems viewpoint using constraint-based modeling allows for simulation of the global effect of genetic modifications.

**Results:**

We present the first genome-scale metabolic model (*i*CM925) for *C. beijerinckii*, containing 925 genes, 938 reactions, and 881 metabolites. To build the model we employed a semi-automated procedure that integrated genome annotation information from KEGG, BioCyc, and The SEED, and utilized computational algorithms with manual curation to improve model completeness. Interestingly, we found only a 34% overlap in reactions collected from the three databases--highlighting the importance of evaluating the predictive accuracy of the resulting genome-scale model. To validate *i*CM925, we conducted fermentation experiments using the NCIMB 8052 strain, and evaluated the ability of the model to simulate measured substrate uptake and product production rates. Experimentally observed fermentation profiles were found to lie within the solution space of the model; however, under an optimal growth objective, additional constraints were needed to reproduce the observed profiles--suggesting the existence of selective pressures other than optimal growth. Notably, a significantly enriched fraction of actively utilized reactions in simulations--constrained to reflect experimental rates--originated from the set of reactions that overlapped between all three databases (*P *= 3.52 × 10^-9^, Fisher's exact test). Inhibition of the hydrogenase reaction was found to have a strong effect on butanol formation--as experimentally observed.

**Conclusions:**

Microbial production of butanol by *C. beijerinckii *offers a promising, sustainable, method for generation of this important chemical and potential biofuel. *i*CM925 is a predictive model that can accurately reproduce physiological behavior and provide insight into the underlying mechanisms of microbial butanol production. As such, the model will be instrumental in efforts to better understand, and metabolically engineer, this microorganism for improved butanol production.

## Background

The diminishing supply of non-renewable feedstocks--and concern over environmental ramifications of their use in fuel and chemical production--highlights the need for technological advances to improve the economic viability of sustainable production methods. In particular, given its broad scope of applications as a chemical feedstock and compelling properties as an alternative transportation fuel [1], sustainable production of butanol is of particular industrial interest. Butanol production via microbial fermentation from lignocellulosic material (historically achieved using solventogenic clostridia, prior to petroleum refining [2]) represents a sustainable method for production of this important solvent.

Recently, the most common fermentation microorganisms--*Escherichia coli *and *Saccharomyces cerevisiae--*have been engineered to produce butanol and its branched chain derivatives (e.g., isobutanol) [3-5]. However, relative to *E. coli *and *S. cerevisiae*, the solventogenic clostridia offer two clear advantages as butanol-producing microorganisms: (i) the evolved ability to produce and tolerate butanol at concentrations up to 21 g/L--important because butanol is highly toxic to microorganisms at even low concentrations [6-9], and (ii) the ability to co-ferment pentose and hexose sugars, the primary sugars found in lignocellulosic hydrolysates [10,11]. These characteristics should reduce the number of genetic modifications needed to make biological butanol production economically competitive.

Among the solventogenic clostridia, *Clostridium acetobutylicum *ATCC 824 and *C. beijerinckii *produce the highest n-butanol concentrations; the mutant strain *C. beijerinckii *BA101 achieves the highest reported concentration (17-21 g/L) across all microorganisms [7-9]. The parent strain of BA101, *C. beijerinckii *strain NCIMB 8052, holds several advantages for industrial butanol production: (i) it has proven amenable to experimental modifications that increase butanol tolerance and production [9]; (ii) the solventogenic genes reside on the chromosome rather than on a separate megaplasmid (as is the case for *C. acetobutylicum*), potentially increasing its resistance to degeneration [12]; (iii) it can successfully produce butanol in continuous culture conditions [13]; and (iv) it has a broad substrate utilization spectrum [10,11,14]. Taken together, these traits give *C. beijerinckii *particular appeal as a clostridial catalyst for industrial butanol production.

Like other solventogenic clostridia, *C. beijerinckii *produces solvents (butanol and acetone) as products of a biphasic metabolism. Butyrate and acetate are produced first in acidogenesis; in solventogenesis, acids are re-assimilated and production of butanol and acetone begins. Central to improving butanol production is deciphering what causes this metabolic switch. Numerous phenomena--decreased pH, acid accumulation, intracellular ATP concentration, nutrient limitation, interplay between carbon and electron flow pathways, and sporulation--have been hypothesized to contribute [15,16]. Most of these phenomena are directly tied to cellular metabolism, or more specifically, to changes among the enzymes and metabolites that comprise the intracellular metabolic network. Our aim is to develop an understanding of *C. beijerinckii *metabolism--through systems analysis of the metabolic network--that will enable us to optimally direct available carbon towards the production of butanol.

*In silico *reconstruction of metabolic networks--and subsequent analysis of genome-scale metabolic models through constraint-based modeling [17]--enables a global interrogation of metabolism not possible with standard experiments. The genome-scale model allows one to analyze the cell from a systems viewpoint to predict whole-cell effects of genetic changes, and to simulate known and hypothesized phenotypes. Methods for reconstructing and analyzing metabolic networks have been well established for microorganisms, and genome-scale models have been built for all branches of life [18,19]. Furthermore, numerous successes have been demonstrated for using these models to guide rational engineering in model microorganisms such as *E. coli *and *S. cerevisiae *[20-22]. Importantly, models of this type can be constructed for any organism with a sequenced genome, and thus hold particular utility for lesser-characterized organisms such as *C. beijerinckii*.

We present here the first genome-scale metabolic model (named *i*CM925) for *C. beijerinckii*, built based on the NCIMB 8052 strain. There have been four genome-scale models built for clostridia--two for *C. acetobutylicum *[23-25] and one each for the cellulolytic strains *C. thermocellum *[26] and *C. cellulolyticum *[27]. *C. beijerinckii *is distinct from these in that it is the most productive wild-type butanol-producing clostridia known to date [6-9]. Containing 925 genes, 938 reactions, 881 metabolites, and 67 membrane transport reactions, *i*CM925 is the largest genome-scale model for a clostridial species. The *i*CM925 model can simulate substrate uptake and product formation rates for typical batch culture experiments, and correctly captures the relationships between the formation of products such as butanol and hydrogen. As such, the *C. beijerinckii *model will be instrumental in our future efforts to engineer *C. beijerinckii *to produce higher titers of butanol.

## Results & Discussion

The first step towards building a genome-scale metabolic model is reconstructing the genome-scale metabolic network--typically done using publically available annotation databases and published literature. A list is collected of reactions that are either catalyzed by enzymes encoded in the genome or have been defined experimentally, and then expanded to define relationships between genes, enzymes, reactions, metabolites, and pathways in the network. To establish the genome-scale metabolic model, the network reactions are subjected to a number of physico-chemical constraints--either calculated or based on physiological data--to simulate defined cultural conditions. Given the limited literature and biochemical data available for *C. beijerinckii*, we reconstructed our metabolic network first using a semi-automated approach to obtain annotation data from three major databases, and then utilized computational algorithms and manual curation to further refine the network. To test the ability of the *i*CM925 model to simulate experimentally-observed behavior, we conducted a series of batch fermentations to compare measured substrate uptake and product formation rates with model predictions. The model provides a solid basis with which to study the unique characteristics of *C. beijerinckii *metabolism and guide future metabolic engineering experiments for enhanced butanol production capability.

### The initial genome-scale metabolic network

The available genome annotations for lesser-characterized organisms are largely generated by computational, informatics-based procedures (i.e., they often lack manual curation), and there is a paucity of experimentally-confirmed biochemical data. To facilitate reconstruction, expand the scope of our *C. beijerinckii *network, and evaluate confidence for each gene-protein-reaction (GPR) relationship included, we merged annotation data from three independent databases: KEGG (Kyoto Encyclopedia for Genes and Genomes) [28], BioCyc [29], and The SEED [30,31]. To reduce the time required to assemble annotation data into a well-connected genome-scale network, we employed a semi-automated computational approach (see Methods) to retrieve and integrate information from each database.

The foundation for our network, comprising 525 reactions, was obtained from the KEGG database. We expanded this network to include an additional 75 and 136 unique reactions from The SEED and BioCyc databases, respectively. Careful reconciliation and integration of the obtained biochemical data was required because the three databases do not follow a uniform nomenclature for reactions, metabolites, and pathways. We chose to adhere to the nomenclature used by the BiGG database (the largest available repository for genome-scale metabolic models) in order to enable easier comparison with other *in silico *models [32]. This mapping step was quickly accomplished by using a matrix formalism to overlay the different databases (see Methods) based on stoichiometry. The mapping between BioCyc and KEGG for reaction and metabolite names in *C. beijerinckii *is available in Additional File 1.

**Figure 1:**
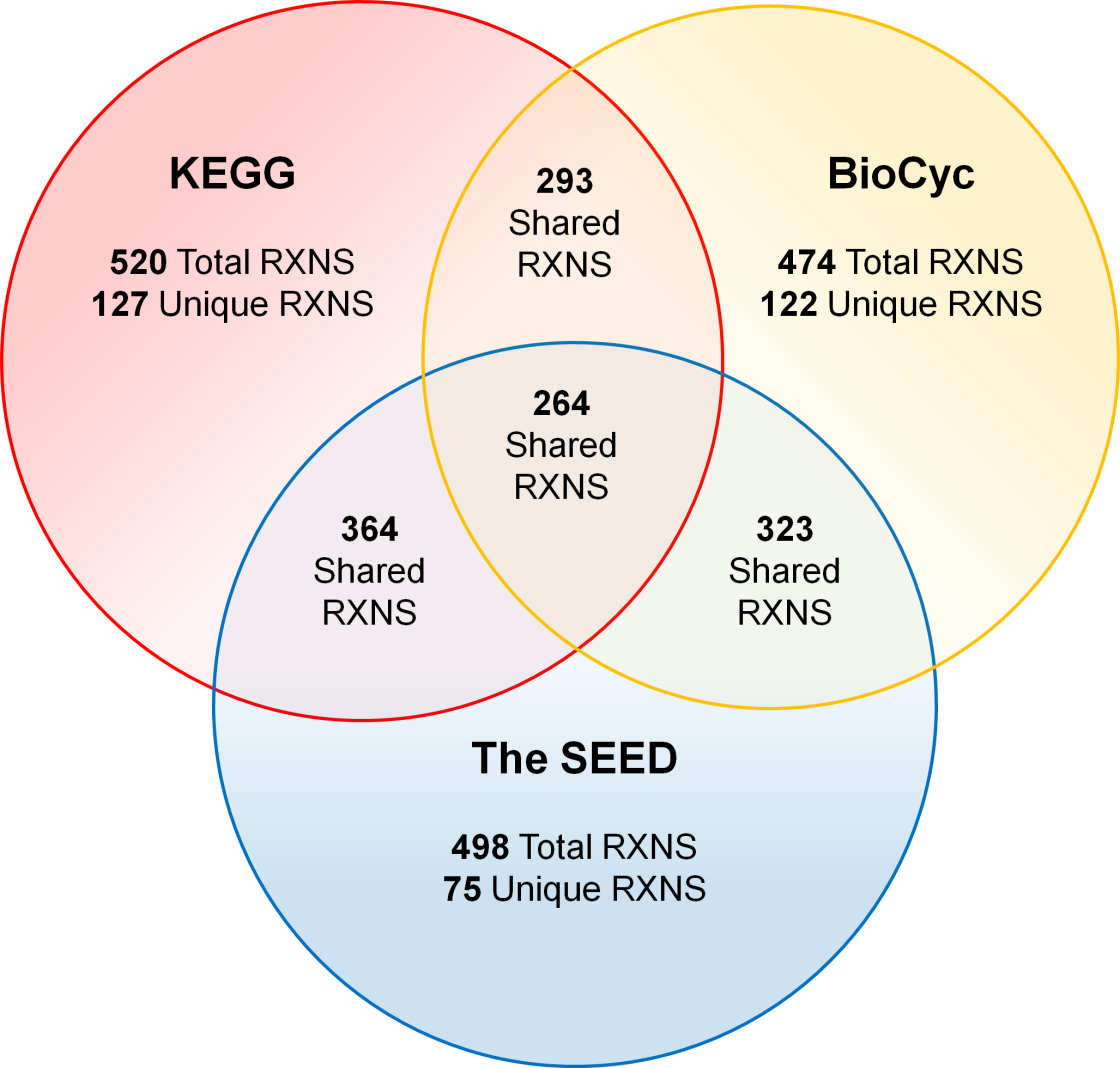
**Annotation database comparison**. Reaction overlap for the three annotation databases used to build the genome-scale metabolic network.

We analyzed the overlap between annotation information collected from KEGG, BioCyc, and The SEED to help assess the reliability of each reaction included in the network. Reactions found in all three databases were considered to have the greatest reliability, followed by reactions in two of the databases, and finally by reactions found in only one database. Surprisingly, out of the collective 776 suggested reactions, we found only 264 reactions (34%) present in all three annotations (Figure 1). Given that many genome-scale models are built in a similar manner, the small overlap observed for *C. beijerinckii *suggests that researchers must exercise caution when constructing networks for new organisms using bioinformatics-based annotations alone. The reconstruction and phenotypic testing of genome-scale models provides an important means to integrate, curate, and validate annotation information. Further analysis of the relationship between database contribution and model accuracy (used to evaluate annotation quality for *C. beijerinckii*) is discussed below.

In addition to establishing reliability for each included reaction, we evaluated the predicted gene-associations for reactions found in two or more of the annotation databases (see Additional File 2A for database-based GPR comparison). In cases where annotations did not agree between databases, associations were selected for the model based on the strongest BLAST [33] evidence (i.e., genomic identity between the associated enzyme and similarly annotated database proteins). Reassuringly, we found that most annotation disagreements were due to a missing gene-reaction relationship rather than a contrasting association; this suggests that overlapping reactions comprise a well annotated area of the network.

### The refined *C. beijerinckii* metabolic network

The draft metabolic network derived from genome annotation data--even with combined information from multiple databases--contained gaps (i.e., missing reactions) that prevented simulation of cell growth and accurate physiological behavior (e.g., butanol production). Gaps create unconnected sections/regions in the network, thereby preventing production or consumption of a metabolite. In turn, the "dead-end" metabolite has often been observed experimentally as consumed or produced, or is needed to simulate cell growth. Network gaps must therefore be filled using literature information and/or genomic evidence beyond what was included in the annotation databases.

Identifying network gaps and selecting candidate gap-filling reactions with strong supporting evidence can be time consuming, especially for lesser-characterized organisms like *C. beijerinckii*. Consequently, we used the GapFind and GapFill [34] algorithms to computationally identify and resolve gaps, thus minimizing the amount of manual curation needed. Candidate reactions suggested by GapFill were chosen from the BiGG database; this database contains genome-scale models that have undergone extensive refinement and validation, and thus is a resource of high-confidence reactions [32]. After reviewing candidate reactions for sufficient BLAST [33] evidence, we identified an additional 22 putative annotations (and 22 additional network reactions) for the *C. beijerinckii *genome (a list of added reactions may be found in Additional File 1)--seven of which were required for simulated cell growth. While GapFind and GapFill are highly useful computational algorithms, we found that they are not guaranteed to suggest reactions with strong supporting evidence and do not focus specifically on fulfilling the model objective function. Therefore, manual addition of reactions based on literature evidence was still needed to fill important gaps in the *C. beijerinckii *network.

Notably, the draft network was missing a butanol dehydrogenase enzyme, a ferredoxin NAD^+ ^reductase, and did not contain the necessary biochemical transformations for production of known phospholipids. Reactions for both an NAD^+ ^and NADP^+ ^butanol dehydrogenase enzyme (*BUTOHDx *and *BUTOHDy*), known to exist in solvent producing clostridia [2,35], were added based on BLAST [33] scores for the *C. beijerinckii *gene Cbei_2421. We were unable to find a gene association for ferredoxin NAD^+ ^reductase--even though the NADP^+ ^reductase is matched to Cbei_0661 and Cbei_2182--but added the reaction (*FDXNRx*) based on literature evidence [2]. The phospholipid pathway was characterized using a similar approach to Lee et al. [23], drawing upon experimental data for fatty acid biosynthesis [36]. In total, 38 reactions were added as a result of our manual curation--11 of which were added based on BLAST [33] comparison with reactions from the Senger & Papoutsakis *C. acetobutylicum *model [24,25] and 22 of which were added for the formation of phospholipid and biomass components. The source for manually added reactions (as well as all other reactions included in the model) can be found in Additional File 1).

One of the most significant gaps in the draft *C. beijerinckii *network prevented model-simulated production of oxoglutarate, a major component of central metabolism; this gap stemmed from missing genetic evidence for enzymatic reactions need to complete the TCA cycle. We completed the TCA cycle in the model based on conclusions from two recent experimental studies, in which carbon labeling showed that *C. acetobutylicum *uses a bifurcated TCA cycle culminating in succinate secretion [37,38]. The initial reconstruction did not support a bifurcated TCA cycle: our network was missing a citrate synthase (*CS*), succinyl-CoA synthetase (*SUCOAS*), and a succinate transport (*SUCCex*) reaction. In addition, the directionality of existing reactions did not support the experimentally observed flux. To allow for simulation of the bifurcated cycle and enable oxoglutarate production, we added the three missing reactions (without genetic evidence), and restricted reaction directionality to that observed in the study.

### The genome-scale model (*i*CM925)

From our refined metabolic network, we constructed the genome-scale model by representing reactions, gene associations, pathway information, and reaction directionality in matrix form (see Additional File 1 and Additional File 3 for model files). This model for *C. beijerinckii*, named *i*CM925 in accordance with the model naming convention proposed by Reed et al. [39], contains 938 reactions, 881 metabolites, and 925 genes--representing 18% of total protein coding genes in the genome [40,41]. Transport reactions across the cell membrane--collected from the BioCyc and KEGG databases, as well as from the published *C. acetobutylicum *models [23-25] and the genome-scale models for *Bacillus subtilis *[42,43]--make up 67 of the 938 reactions. *i*CM925 contains the largest number of genes, reactions and metabolites compared to the four other clostridial models (Table 1); this could be a result of model construction methods, but likely reflects the fact that *C. beijerinckii *has a 50% larger genome than the other clostridia.

**Table 1:** Network statistics for genome-scale models of clostridia

	*C. beijerinckii* *i*CM925	*C. acetobutylicum* **(Lee)**[23]	*C. acetobutylicum* **(Senger)**[24,25]	***C. cellulolyticum *** [27]	*C. thermocellum* ***i*SR432 **[26]
**Genome**	6.0 Mb	4.1 Mb	4.1 Mb	4.1 Mb	3.8 Mb
**Protein Coding Genes**	5100	3748	3748	3488	3236
**Model Genes**	925	432	474	431	432
**Reactions**	938	507	552	621	577
**Metabolites**	821	479	422	603	525

Statistics for each published genome-scale clostridia model and comparison with *i*CM925.

**Figure 2:**
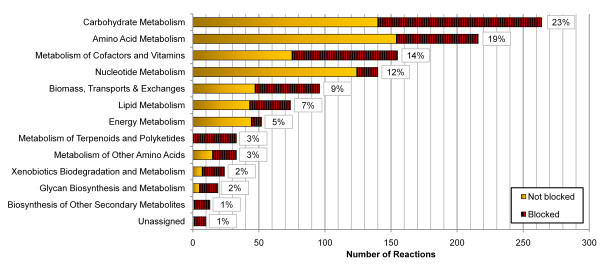
**Pathway distribution for reactions in *i*CM925**. The number of reactions that can carry flux is depicted in yellow and the number that cannot carry flux is depicted in red (with black stripes) for each area of metabolism. Percentages indicate overall percent contribution of that pathway to the model. Blocked reactions were determined by simulating growth on glucose minimal media.

The reactions in *i*CM925 span 95 pathways (organized into 13 major groups in Figure 2), as defined by KEGG pathway nomenclature. Carbohydrate and amino acid metabolism represent the largest portions of the network. For each pathway, we calculated the percentage of reactions that can or cannot be utilized in glucose minimal media simulations (Figure 2) in order to evaluate model connectivity. Overall, 47% of reactions across all pathways are blocked, which is on par with other *in silico *genome-scale models [44]. Many of these blocked reactions were concentrated within pathways that are almost entirely blocked, such as metabolism of terpenoids and polyketides--suggesting that many of the blocked reactions are actually a result of blocked pathways. Pathways involving carbohydrate metabolism (compared to a more connected pathway such as nucleotide metabolism) may have a higher number of blocked reactions under glucose media conditions because they contain numerous reactions intended for metabolism on alternative sugar substrates.

We assessed the pathway contribution of each annotation database (see Additional File 2B ) to determine (i) if any database exhibited more complete coverage in one area of metabolism (e.g., carbohydrate metabolism) and (ii) if one database contributed more blocked reactions to the model. For each of the 13 pathway categories depicted in Figure 2, we found similar coverage between KEGG, BioCyc, and the SEED; this indicates that the small overlap found between databases is not simply a result of one database contributing more heavily to a particular area of metabolism. Additionally, each database contributed a similar number of blocked reactions: 22% of the blocked reactions came from BioCyc, 21% from KEGG, 10% from The SEED, 18% from two or more databases, and 17% from all three databases (these percentages are not directly proportional to the total number of enzymes contributed by each database). Therefore, we did not find that one database outperformed another in terms of model connectivity.

### Validation of *i*CM925

To evaluate the predictive accuracy of *i*CM925, we used Flux Balance Analysis (FBA, see Methods) to reproduce experimental fermentation behavior. The FBA formalism represents all known reactions in the cell as a stoichiometric matrix, and uses linear programming to maximize a user defined objective function (e.g., growth) under a steady state assumption [45,46]. Importantly, FBA can be used to simulate experimental parameters such as growth rates, uptake rates, and byproduct secretion rates--enabling quantitative evaluation of model agreement with physiological behavior.

During fermentation, *C. beijerinckii *produces six primary carbon-containing byproducts: acetate, butyrate, acetone, butanol, ethanol, and carbon dioxide. Due to the biphasic nature of *C. beijerinckii *metabolism, not all five byproducts are produced at the same rates throughout fermentation. In a targeted gene expression study, Shi and Blaschek found that solvent formation began during mid-exponential growth (7-8 hours); this period was characterized by increased expression levels of solvent formation genes and accompanied by decreased expression of genes associated with acid formation [15]. To validate the ability of the model to simulate byproduct secretion and growth rates, we conducted our own batch fermentation experiments using NCIMB 8052 cultures grown on minimal media. Similar to Shi and Blaschek, we observed the switch from butyrate to butanol formation at 8-10 hours; we chose to focus our simulations on the subsequent period of exponential growth in which butanol is produced.

We determined substrate uptake and product secretion rates for cultures grown at four temperatures (30°C, 33°C, 35°C, 40°C) to obtain multiple data sets with which to compare model simulations. Only results for 35°C are reported in the main text, as it is most representative of typical fermentation conditions (complete experimental results are available in Additional File 2C). Experimental rate estimates (in units of mmol/gDW/hr) were determined for butanol, acetone, ethanol, acetate and butyrate using product concentration and growth rate (see Methods, Additional File 2D). We observed a net consumption of glucose and acetate (the carbon containing compounds in our defined growth media) and net production of acetone, butanol and ethanol. When performing simulations, specified uptake and secretion rates were constrained to fall within one standard deviation of experimentally measured rates, while the remaining rates were determined by FBA. All model simulations were conducted with biomass production (defined by the biomass equation, see Methods and Additional File 1 for details) as the assumed cellular objective.

**Figure 3:**
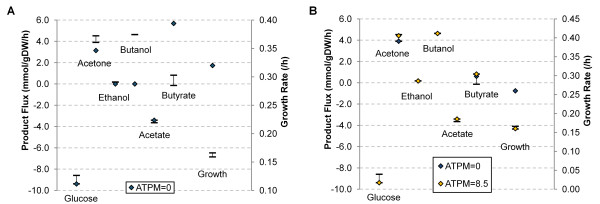
**Comparison of *i*CM925 simulations with experimental data**. Model and experimental values for product fluxes, uptake fluxes and growth rates represent conditions for the 35°C fermentation. Error bars indicate the observed experimental range and diamonds represent the various simulation results. (**A**) shows the simulation results for the case where only acetate and glucose uptake rates are constrained. (**B**) shows the case where these uptake rates, as well as butanol, acetone, and butyrate formation rates are constrained. In (**B**), the blue diamonds represent the case where non-growth associated ATP maintenance is zero and the yellow diamonds represent the case where the non-growth associated ATP maintenance is 8.5 mmol/gDW/hr.

To evaluate model predictions for product formation rates and growth rate we conducted simulations with constrained model uptake of glucose and acetate. For these simulation conditions, the *i*CM925 model predicted production of only acetone and butyrate--production of butanol and ethanol was not predicted (Figure 3A, see Additional File 2D for comparisons at different temperatures). Additionally, the predicted growth rate was higher than our experimentally observed growth rate. These predictions are not surprising for the assumed optimal growth objective, given the experimentally supported understanding of cellular redox in *C. beijerinckii*. Specifically, disposal of excess electrons is achieved in cell culture through the generation of butyrate, butanol, and hydrogen. However, disposal via hydrogen and butyrate would allow for ATP production with minimal loss of carbon, thereby improving the biomass objective. Thermodynamic limitations of the hydrogenase reaction prevent such disposal biologically [2,47], but such constraints were not incorporated into *i*CM925 explicitly because clear experimental hydrogen formation rates were unavailable. Acetone production in the model could also be traced to acetate re-uptake: the formation of acetone utilizes acetoacetate, a byproduct of acetate re-uptake by CoA-transferase.

To confirm that *i*CM925 is capable of simulating production of all expected metabolites at experimentally determined rates, additional constraints were added to the product secretion reactions for butanol, acetone, ethanol, and butyrate (Figure 3B, see Additional File 2D for comparisons at different temperatures). As product formation is known to be associated with the generation of ATP in the cell [2], the effect of ATP production requirements was analyzed by altering the constraints on the non-growth associated ATP maintenance (*NGAM*) reaction. The first simulation assumed that no ATP is needed for non-growth associated maintenance, and resulted in a higher growth rate than expected; this is a biologically unrealistic assumption, but illustrates the dependency of growth rate on ATP maintenance. The latter simulation--with an *NGAM *value that guided the *in silico *growth rate to the experimentally calculated range--demonstrated that the expected experimental phenotype can be reproduced by the model. The selected *NGAM *value was 8.5 mmol/gDW/hr, which is encouragingly similar to the value used in the *E. coli i*AF1260 model [48]. From these simulations, we concluded that all observed secretion patterns exist within the solution space of the model, even though solvent secretion patterns in *C. beijerinckii *are not very well described by the *i*CM925 model when using the optimal growth objective.

### Analysis of the active reactions in *i*CM925

**Figure 4:**
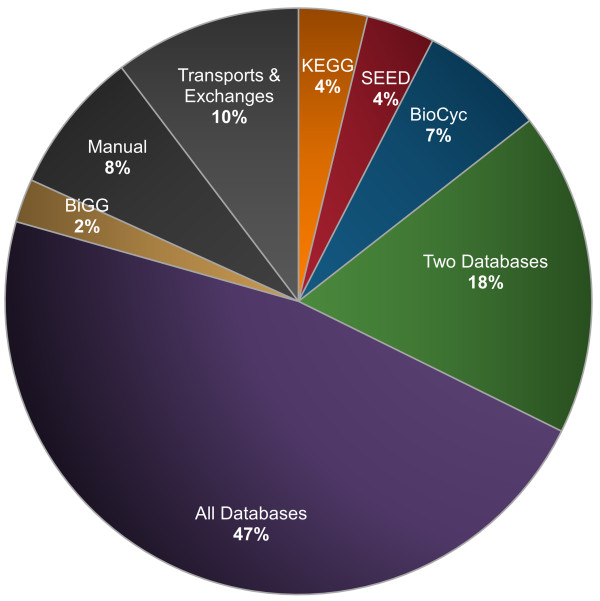
**Origin of active reactions**. Percentages represent the fraction of all active reactions (in the constrained simulation, based on 35°C experiments) originating from individual databases, a combination of databases, or other sources.

After verifying that *i*CM925 could reproduce experimental uptake and secretion rates, we investigated the underlying flux distributions used by the model to achieve these rates. Under optimal growth conditions for a defined minimal medium, a previous study found that genome-scale models for *Helicobacter pylori, Staphylococcus aureus, E. coli*, and *S. cerevisiae *have about 300 active reactions [44]; *i*CM925 had 291 active reactions. Interestingly, 137 of these 291 reactions (Figure 4) were found in all three annotation databases, representing a statistically significant number of active reactions among the overlapping reactions (*P *= 3.52 × 10^-9^, Fisher's exact test; see Methods). Since active reactions are those used by the model to reproduce known physiological behavior, the over-representation of reactions found in all three databases supports our assumption that overlapping reactions have the highest reliability for inclusion.

**Figure 5:**
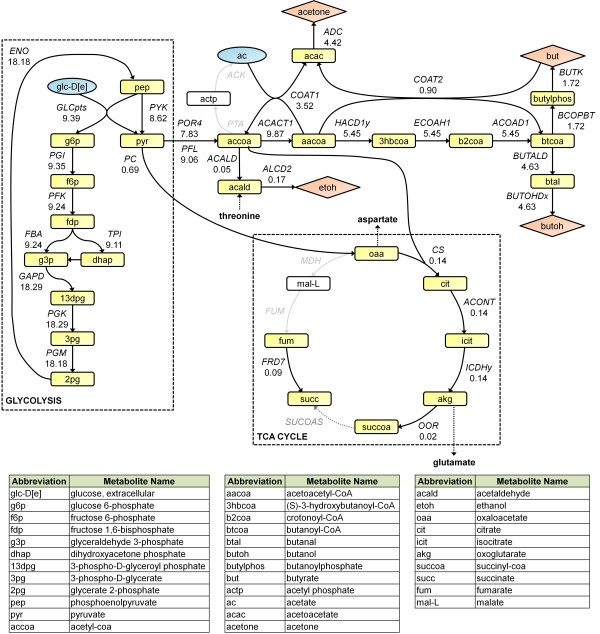
**Network map of important active reactions**. Network fluxes were determined in 35°C fermentation simulations with ATPM = 8.5 mmol/gDW/hr. Blue ovals indicate substrates, red colored diamonds indicate products, and yellow boxes indicate intracellular metabolites only. Numbers next to each reaction name represent flux predicted by the model. Fluxes are not necessarily consistent from one reaction to the next because other, smaller flux pathways have interplay with the reactions here.

To further study active reactions, we diagramed reactions carrying the largest flux in glycolysis, TCA cycle, and the product formation pathways (Figure 5). In glycolysis, we found that the model used the PTS rather than ABC transporter to uptake extracellular glucose. The choice of PTS over ABC suggests that *C. beijerinckii *may use the former transporter primarily as the most efficient means of converting glucose to biomass, a finding that is corroborated by experimental observation of PTS transport (*GLCpts*) utilization by *C. beijerinckii *[14,16,49]. Flux through the TCA cycle follows the experimentally observed route of oxoglutarate production [37] via citrate synthase (*CS*). However, the model did not utilize the oxaloacetate to succinate transformation or the conversion of succinyl-CoA to succinate, as was observed by Amador-Noguez et al. For succinyl-CoA synthetase (*SUCOAS*, an ATP generating reaction), we found that increased ATP requirements resulted in activation.

Contrary to experimental ethanol production which stems primarily from acetyl-CoA [2,40], *i*CM925 predicted that about 70% of ethanol was made from threonine (derived from aspartate) by the enzyme threonine acetaldehyde-lyase (*THRA*). Butanol, butyrate, and acetone were produced using the experimentally characterized pathways, and acetate was consumed using CoA-transferase (*COAT1*) as expected [2,40]. Intriguingly, the model predicted simultaneous production and consumption of butyrate using butyrate kinase (*BUTK*) and CoA-transferase (*COAT2*), respectively. The re-uptake of acids by solventogenic clostridia has been experimentally established, with one of the leading suggestions for this behavior being a means of de-toxification of the acidic environment [2]. Given that the primary objective in our simulations is to maximize flux through the biomass equation within the imposed constraints, it is most likely that the motivation for re-uptake of butyrate by the *i*CM925 model is the generation of additional ATP--a major component of biomass. Previous experimental studies investigating acid re-uptake [41-43] do not support this suggested motivation, however--making additional exploration into the motivations of the model an interesting area of focus going forward. These experimental findings further suggest that selective pressures other than optimal growth may dominate phenotype under typical fermentation conditions.

**Figure 6:**
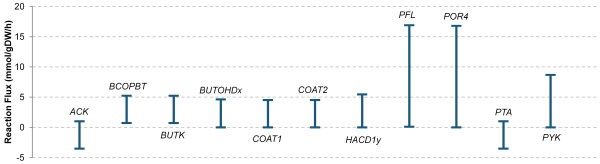
**Flux Variability Analysis of important active reactions**. Bars depict the possible range (minimum and maximum) fluxes calculated by Flux Variability Analysis for reactions depicted in **Figure 5**.

Flux Variability Analysis (FVA) was performed to evaluate the robustness of our diagrammed reactions (Figure 6, see Additional File 2E for a complete list). FVA calculates the extent to which network reactions can change without affecting the simulated maximal growth rate--the model represents an underdetermined system, and even when optimizing for a specific objective, multiple solutions exist for each set of constraints [50]. *ACK, PTA, BUTK, BCOPBT, COAT1*, and *COAT2 *are connected to the uptake and production of acetate and butyrate. As suspected, it is possible for both metabolites (either together or independently) to be simultaneously produced and consumed. The variation seen in *BUTOHDx *and *HACD1x *indicates that either the NAD^+ ^or NADP^+ ^versions of these reactions can be used with no effect on growth rate. Similarly, the variation observed in *PFL, POR4*, and *PYK *shows equally optimal methods of pyruvate formation and consumption.

### Understanding butanol production: the role of molecular hydrogen

**Figure 7:**
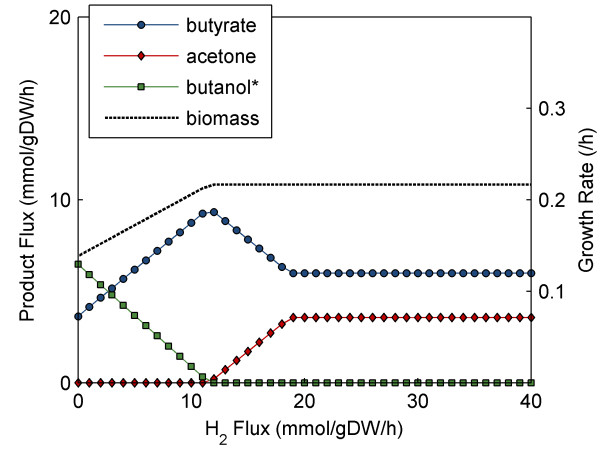
**Effect of hydrogen formation rate with fixed glucose and acetate uptake**. H_2 _output flux was varied to examine the effect of hydrogen production on predicted formation rates for butyrate, acetone, butanol, ethanol, and biomass. Glucose and acetate uptake rates were fixed to 9.39 and 3.41 mmol/gDW/hr, respectively, and non-growth associated maintenance was set to 8.5 mmol/gDW/hr. Note that while positive ethanol formation is not depicted in the plot, FVA found ethanol and butanol production to be interchangeable, with no detrimental result to growth rate--likely because the net consumption of NADH is identical in both scenarios. Experimentally, ethanol formation happens at a slower rate than butanol formation.

Hydrogen formation is known to play an important role in balancing cellular redox for *C. beijerinckii*, and has been found to effect the production of butanol [2,51,52]. We confirmed this relationship in the *i*CM925 model using robustness analysis [17,50] to compare the effects of varied hydrogen secretion rate on the production of acetate, butyrate, acetone, butanol and ethanol. When grown on glucose and acetate, we found that maximizing the specific growth rate led to the formation of acetone and butyrate only; this simulation had a predicted hydrogen production rate of about 18 mmol/gDW/hr (Figure 7). Our analysis showed that in order to observe positive butanol production, hydrogen production must be limited to below about 10 mmol/gDW/hr; the corresponding growth rate becomes sub-optimal with this constraint. Since the production mechanisms of ethanol and butanol both consume the same number of NADH molecules (two in each pathway), the model predicted that at low hydrogen production rates, either ethanol or butanol generation could be used to balance redox with no change in growth rate. *In vivo*, regulation likely plays a role in determining how much of each product is made.

**Figure 8:**
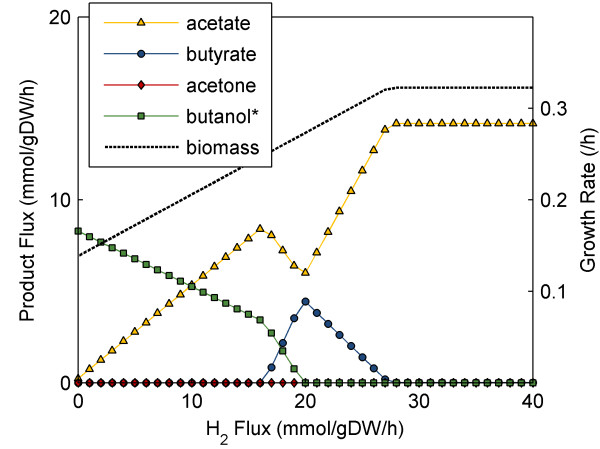
**Effect of hydrogen formation rate with fixed glucose uptake only**. H_2 _output flux was varied to examine the effect of hydrogen on the production of acetate, butyrate, acetone, butanol, ethanol and biomass for optimal growth simulations on only glucose, with an uptake rate of 9.39 mmol/gDW/hr and a non-growth associated ATP maintenance of 8.5 mmol/gDW/hr. Note that while positive ethanol formation is not depicted in the plot, FVA found ethanol and butanol production to be interchangeable with no detrimental result to growth rate.

The tradeoff observed in our simulations between hydrogen formation and solvent formation has been experimentally observed in *C. acetobutylicum*. Kim, et al. [52] found that a decrease in hydrogenase activity (induced by carbon monoxide poisoning) when grown on glucose resulted in decreased growth rate, decreased acetone, acetate, and butyrate production, and increased ethanol and butanol production. As this experiment was conducted without acetate in the initial media, we again investigated the effect of hydrogen production rates, but with glucose as our only carbon-containing model input (Figure 8). We observed that lower hydrogen formation rates coincided with higher solvent formation, as was found by Kim et al. [52] and in our simulations using both acetate and glucose as inputs (Figure 7)--suggesting that similar mechanisms may be involved in butanol production by both *C. beijerinckii *and *C, acetobutylicum*.

Additionally, our simulation shows that for high levels of hydrogen formation, acetate is the only byproduct and growth rate is at a maximum. The production of hydrogen eliminates the need for additional NADH consumption by butyrate, allowing ATP generation to occur exclusively via acetate formation--the most efficient method for the cell. Maximum growth rate is observed under these conditions because they represent the most energy efficient means of glucose utilization for the microorganism. At very low hydrogen consumption rates, we observe production of butanol rather than butyrate, as the production of butanol results in the consumption of two additional NADH molecules compared to butyrate. This observation supports the conclusion that without hydrogen production, excess electrons are disposed of via the production of acids and solvents. The overall observed effect of hydrogen formation is not only experimentally consistent, but it highlights the importance of this reaction in regulating butanol formation, and will be an area of focus in our continued efforts to improve butanol production in *C. beijerinckii*.

### Comparison of *i*CM925 with *C. acetobutylicum* model

**Figure 9:**
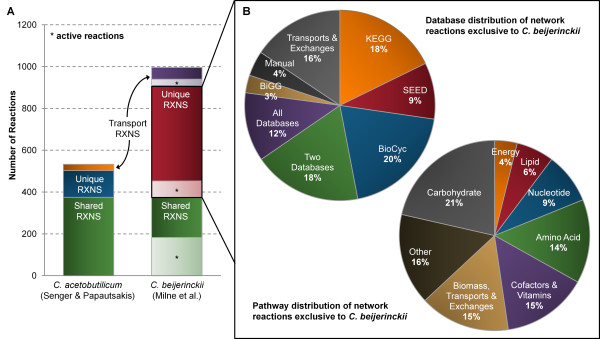
**Comparison of *i*CM925 with the Senger & Papoutsakis *C. acetobutylicum *model**. The number, database distribution, and pathway distribution of reactions in *i*CM925 and the Senger & Papoutsakis *C. acetobutylicum *model were compared based on KEGG IDs. (**A**) Numbers of reactions in common between the two models or unique to each are depicted by bars on the graph. The fractions of unique and shared reactions that are active in *i*CM925 are denoted by light-shaded regions. (**B**) The database distribution of reactions exclusive to *i*CM925 are shown in the upper left, while the pathway distribution is shown in the bottom of the panel.

Although the genome of *C. beijerinckii *is 50% larger than that of *C. acetobutylicum*, the two microorganisms present phenotypically similar fermentation profiles. To investigate the effect of additional genes in *C. beijerinckii*, we compared *i*CM925 to the *C. acetobutylicum *model which was published in a computable format (the model published by Senger and Papoutsakis in 2009 [24,25]), using KEGG reaction IDs as a basis for comparison. Of the 940 *i*CM925 reactions, 375 were found to overlap with the Senger model (Figure 9); 183 of these reactions are present in our list of 291 active reactions for the 35°C (*ATPM *= 8.5 mmol/gDW/hr) fermentation simulation. Interestingly, the pathways and database sources of the 564 reactions unique to *i*CM925 were similarly distributed as those of the full model--suggesting that (i) *C. beijerinckii *does not simply contain more reactions in a particular pathway, and (ii) that our additional reactions are not an artifact of our multiple database approach. Of the 375 overlapping reactions, 119 have more connected genes per reaction in *C. beijerinckii *than in *C. acetobutylicum*--with an average of 1.3 times more genes per reaction in *C. beijerinckii*. This is not a statistically significant result, but suggests that several of the reactions (e.g., CoA-transferase and butyrate kinase) do have more associated genes than the corresponding *C. acetobutylicum *reactions.

## Conclusions

Butanol, currently produced as a byproduct of petroleum refining, is appealing in industry as both an important chemical feedstock and an alternative transportation fuel. We have built the first genome-scale model for *C. beijerinckii *to better understand the metabolic behavior of the microorganism, and to guide future metabolic engineering for increased butanol production. Having a genome-scale model for *C. beijerinckii *is advantageous because it helps provide a global picture of metabolism--enabling interrogation of the interplay between the various fermentation products of the microorganism from a systems viewpoint. Given the lack of detailed biochemical data available for *C. beijerinckii*, we integrated and cross-checked information from three major annotation databases to reconstruct the core metabolic network, and then further completed the network with computational algorithms and manual curation. We collected experimental fermentation data to determine production rates of acetone, ethanol, and butanol, and uptake rates of acetate and glucose, and these rates were used to confirm the ability of the model to accurately represent physiological behavior. Interestingly, reactions found in all three annotation databases proved to contribute significantly to the actively used reactions in validation simulations. Even though the observed experimental phenotypes were found to exist in the solution space of the model, optimal growth simulations on glucose did not predict the expected product profiles--suggesting the possibility of an alternative cellular objective or additional mechanisms not captured by the *i*CM925 model. One reaction found to have a strong impact on the predicted product formation rates was the hydrogenase reaction--a reaction that has been found to impact solvent formation experimentally as well. Going forward, this model will play a central role in understanding and engineering butanol production by *C. beijerinckii*.

Additionally, the construction of *i*CM925 highlighted important areas of investigation for future model-building efforts in other lesser-characterized microorganisms (e.g., genome annotation agreement), and for improved constraint-based simulation of non-growth phenotypes (e.g., alternative objective functions).

## Methods

Genome-scale models are built using enzyme-catalyzed reaction information encoded in the genome of an organism, as well as experimentally characterized reaction information. This collection of all known reactions in the metabolism of the organism then serves as the foundation for the metabolic model. The model is represented mathematically by the stoichiometric (**S**) matrix. Each column in **S **represents a reaction in the network, where entries for each row indicate the stoichiometric relationship of corresponding metabolites (negative and positive coefficients denote reactants and products, respectively, and zero entries indicate a non-participating reaction). Through constraint-based modeling [17,53], a series of balances and bounds (discussed below) are applied to the reactions in **S**, and the model is used to simulate cell growth by optimizing for a user-defined objective function. These simulations can then be used to examine the interplay between different reactions and pathways, and to predict resulting metabolic phenotypes from genetic modifications.

### Semi-automated compilation of the draft metabolic network

The metabolic network describes the connectivity of metabolites and reactions in a cell and characterizes the link between genes, proteins and reactions (GPR relationship). We built the base metabolic network using the KEGG genome annotation for *C. beijerinckii*. This draft network contained annotation-based information available for *C. beijerinckii *from the KEGG database, including GPR relationships, pathway information, reaction stoichiometry, and reaction reversibility. To reduce the time needed to generate the initial draft network we developed a MATLAB based program to automatically collect and organize organism-specific biochemical information from the KEGG database.

The KEGG draft network was augmented using independent annotations from The SEED and from BioCyc. Like with the KEGG database, these annotations were collected and organized automatically using MATLAB. Additionally, we used MATLAB to automate the integration of all three databases as much as possible. Annotations in The SEED database are linked to KEGG biochemical data, making integration of the two networks straightforward. BioCyc, however, employs a different nomenclature, so we constructed a mapping between reaction and metabolite IDs in BioCyc and KEGG for *C. beijerinckii*. Specifically, metabolites were mapped using (i) BioCyc files linking to KEGG (incomplete); (ii) compound names and unique iNICHi identifiers; (iii) the *E. coli *specific mapping for *i*AF1260 [48]; and (v) manual curation. Between-database reaction mapping was then determined as follows:

i. using the between-database metabolite mapping, we identified the set of all compounds shared by BioCyc and KEGG;

ii. we built temporary **S **matrices (one for the BioCyc *C. beijerinckii *reaction set, one for the KEGG/SEED draft network set, and one for the BiGG database--including only those reactions involving metabolites in the shared set (metabolites were ordered identically in each matrix);

iii. we identified matching columns (reactions), assuming that identical stoichiometric relationships between a set of metabolites represented a matching reaction between BioCyc and KEGG.

iv. we manually inspected and curated reactions not mapped in an automated fashion.

The resulting network, based on KEGG nomenclature, consisted of the list of reaction formulas, the corresponding enzymes (including enzyme commission number) and genes, reaction identifiers, pathway information, and a note on the source database. To enable comparison with other published genome-scale models (over 50 to date [21]), the metabolite and reaction identifiers were reformatted in accordance with models available in the BiGG database [32]. This was done similarly to the BioCyc-KEGG mapping described above. Metabolite mapping was achieved using flat files from BiGG, and reactions were mapped using temporary **S **matrices for BiGG and the *C. beijerinckii *network. Manual matching was performed for reactions and metabolites for which no automated connection was found. Names were generated for any remaining reactions and metabolites for which no mapping existed.

After identifying active reactions in defined model simulations (described below), we examined whether there was a strong association between database overlap and inclusion in the active set. Specifically, we used the Fisher's exact probability test to determine whether the set of overlapping reactions (those found in all three annotation databases) was "enriched" for active use in a simulation. Using the '*fisherextest*' function available for MATLAB, we defined a 2 × 2 table representing the following frequencies: (i) reactions belonging to all databases that were active in the simulation; (ii) overlapping reactions that were inactive; (iii) non-overlapping reactions (those belonging to two or less databases) that were active; and (iv) non-overlapping reactions that were inactive in the simulation. An exact P-value--indicating the probability of observing the same or higher frequency of overlapping, active reactions by random chance--was calculated by the function based on a hypergeometric distribution.

### Building the genome-scale metabolic model

In order to simulate cellular behavior based on a defined set of inputs and outputs, the network derived from KEGG, BioCyc, and The SEED was converted into a genome-scale metabolic model. As described above, the stoichiometric matrix (**S**) contains the primary model information. The fundamental equation used to model the system is based on the net mass balance of reactions in the network, defined by:

$$Sv=\frac{\text{dx}}{\text{dt}}$$

where dx/dt is change in metabolite concentration over time and the flux vector *v *represents the rate of biomolecular conversion for each reaction (units of mmol/gDW/hr). Constraint-based modeling [17] typically assumes steady state operation (mass into the cell equals mass out), leading to the following mass balance constraint:

Sv=0

When building the model, application of physico-chemical constraints--namely mass and energy balance--were carefully enforced. To mass- and charge-balance model reactions, charge information for each molecule was determined using (in order): (i) the BiGG database; (ii) computational pKA based predictions at pH 7.2; and (iii) BioCyc (see Additional File 1 for complete list). The model was then mass balanced in a semi-automated fashion using charged molecular formulas. Reactions with a hydrogen imbalance were balanced by automatically altering the stoichiometric hydrogen relationship until both mass and charge balances were satisfied. Reactions that could not be balanced in this manner were inspected manually. Any reactions that ultimately could not be balanced were excluded from the model entirely.

We applied environmental constraints as bounds on individual fluxes (e.g., flux capacities, thermodynamics), defining the smaller solution space that represents the allowable phenotypes for the model. Irreversible reactions were constrained to positive or negative flux, depending on direction. Membrane transport and exchange reactions were used to transfer metabolites into and out of the cytosol and system boundaries, respectively. For metabolites whose uptake or output rates were experimentally determined, we specified individual bounds (e.g., glucose, acetate) on the corresponding exchange reactions, and these were varied depending on the simulation. Reversibility was determined by careful comparison of reaction direction in all databases, and the most common directionality was typically chosen. We used extreme pathway analysis [54,55] to identify thermodynamically infeasible cycles, and eliminated these cycles by changing directionality or deleting one of the participating reactions.

As the constraint-based system is highly underdetermined, many solutions (i.e., flux distributions) exist that satisfy **S***v *= 0. We therefore used Flux Balance Analysis (FBA) to determine the distribution of reaction fluxes that optimize a user-defined biological objective function (in our simulations, the commonly used biomass production objective) [46,56]. To facilitate simulations, the model was formatted to be compatible with the COBRA Toolbox [50]; all model simulations were subsequently performed using COBRAToolbox-1.3.1 in MATLAB, with GLPK as the linear programming solver. For all optimizations, the '*minNorm' *flag was turned on (related to the cost of enzyme production in the cell), and simulations were run with a negative lower bound representing a reversible reaction.

To simulate biomass production, a single equation representing all macromolecules comprising one *C. beijerinckii *cell was created using known experimental compositions and compositions inferred from the genome. Following the detailed supplemental information provided by Lee et al. [23] for their *C. acetobutylicum *genome-scale model, biomass was assumed to consist of: DNA, RNA, lipids, protein, peptidogylcan, and teichoic acid and trace metabolites. DNA, RNA and protein content were calculated directly from the genome sequence, and peptidoglycan, teichoic acid and trace metabolites were kept similar to *C. acetobutlylicum*. To determine the appropriate lipid composition, we performed a detailed analysis of lipid and fatty acid content in the cell using data from [36]. See Additional File 1 for more details.

When performing Flux Variability Analysis [17,50], we selected reactions that could increase or decrease by 25% of their maximal flux value for further analysis. To simulate the effect of hydrogen production on the production of acetate, butyrate, acetone, butanol, ethanol and growth we performed a robustness analysis [17,50]. This was done by constraining the flux through the hydrogen exchange reaction and iteratively performing FBA to evaluate changes in flux through the exchange reactions of other products. The reaction flux through exchange reactions for all interested metabolites was then plotted versus flux through the hydrogen exchange reaction.

### Refinement of the genome-scale metabolic model

As defined by Kumar et al., metabolites that participate in network gaps fall into two categories: non-produced or non-consumed. We first used GapFind/GapFill [34], which identifies network gaps and suggests reactions (from a user-specified database--in our case, the BiGG database) whose addition to the model would eliminate the gap. Suggested reactions were manually inspected for relevancy and homology evidence using BLAST [33]; reactions with an E-value of 1 × 10^-8 ^or less for their associated gene were added to the model. This liberal cut off was used in an effort to achieve biomass growth--the identified gene associations for the added reactions were later compared against KEGG, BioCyc and The SEED. If one of the suggested gene associations was found to have a stronger annotation in one of these databases, the corresponding reaction from GapFill was not added to the model. To identify replacement reactions, we iteratively ran GapFind and GapFill until the suggested reactions all had poor supporting genetic evidence.

Additional model refinement was carried out using reactions described in published *C. beijerinckii *material, as well as the two published *C. acetobutylicum *genome-scale models [23-25]. Reactions added from the *C. acetobutylicum *models were added in the same manner as the GapFill suggestions, with a required BLAST [33] E-value of no more than 1 × 10^-8^. In only a few cases, reactions were added without any genomic evidence, given sufficient literature support for the reaction. Model refinement continued until the model was capable of simulating accurate growth and product formation.

### Experimental data collection & analysis

The four fermentation studies were conducted at different temperatures: 30°C, 33°C, 35°C, and 40°C; each study was run in triplicate. Cultures of *C. beijerinckii *NCIMB 8052 were stored in spore form at 4°C in sterile H_2_O [7]. Spores were heat shocked for 10 minutes at 80°C, immediately transferred into an ice bath for 5 minutes, and inoculated into a 6% glucose filter-sterilized P2YE medium [9,57]. The inoculum was incubated in an anaerobic chamber under N_2_:CO_2_:H_2 _(volume ratio of 85:10:5) atmosphere for 14 hours at 35 ± 1°C. Cell cultures were then transferred into 1 L Sixfors Bioreactors (Appropriate Technical Resources, Inc.) containing 400 mL 6% glucose filter-sterilized P2 medium under anaerobic conditions for a 100 hour total fermentation period. Over this time period, samples were taken every 3 hours for the first 24 hours, 6 hours for the next 12 hours, 12 hours for the next 24 hours, and then every 24 hours for the remainder of the time. For each sample, optical density was measured using a UV-Visible Spectrophotometer (Thermo Scientific BioMate 3) and cell density was calculated using the relationship A_600 _= 1 equivalent to 0.28 mg/mL. Gas chromatography (Agilent Technologies 7890A GC System) was used to quantify acetic acid, acetone, butyric acid, ethanol, and butanol concentrations, and glucose concentration was determined using high pressure liquid chromatography (Agilent Technologies 1200 Series). The pH was recorded throughout the fermentation.

For each fermentation run, substrate uptake or product formation rates were calculated using the following equation [58], and then averaged across each temperature condition:

$$\text{rate}=\frac{\Delta \left[\text{metabolite}\right]}{\Delta \left[\text{biomass}\right]}\mu$$

In this equation, [metabolite] is the metabolite concentration in mmol/L, [biomass] is the cell concentration in gDW/L and μ is the growth rate. The yield Δ[metabolite]/Δ[biomass] was determined by plotting metabolite concentration against biomass concentration. Growth rate was found using an exponential growth fit to the biomass vs. time plots. To test the ability of *i*CM925 to reproduce the experimental rates, an experimental "range" was defined as within one standard deviation above or below the mean. This range was used to constrain the upper and lower bounds of the relevant uptake and output reactions in the model, and the resulting *in silico *growth prediction was compared to the experimental growth rate.

## Competing interests

The authors have filed a provisional patent related to the *C. beijerinckii *metabolic model described herein.

## Authors' contributions

CBM completed the bulk of the metabolic reconstruction, model generation, and related analyses, guided and contributed to experimental data collection, and drafted the manuscript. JAE contributed significant intellectual feedback regarding model building and analysis, assisted with the automated collection of annotation data, and substantially edited the manuscript. RR assisted with the initial metabolic reconstruction and model generation, and the initial experimental fermentation studies. SA optimized the experimental fermentation protocol and assisted with experimental data collection and analysis. PJK provided significant intellectual feedback, and coded and executed gapfilling algorithms. RSS provided invaluable intellectual support regarding model analyses and comparisons to *C. acetobutylicum*, and provided significant edits to the manuscript. YSJ provided experimental expertise and equipment, regular feedback regarding model building and analysis, and significant edits to the manuscript. HPB provided invaluable knowledge of *C. beijerinckii *metabolism, experimental expertise and equipment, regular feedback regarding model building and analysis and significant edits to the manuscript. NDP conceived of the study, guided the project, contributed to the design of the metabolic reconstruction, model generation, and subsequent analyses, and substantially edited the manuscript. All authors read and approved the final manuscript.

## Supplementary Material

Additional file 1 **Model Details**. This Excel file contains detailed information about the following aspects of the *i*CM925 model: • reaction information (**reactionData**). • metabolite information (**metaboliteData**). • reaction additions from BiGG based on GapFill suggestions and BLAST scores (**GapFill additions from BiGG**). • biomass equation and details about its construction (**Biomass Composition**). • fatty acid and phospholipid molecular formulas (**Phospholipid Formulas**). Click here for file

Additional file 2 **Additional Figures and Analyses**. This PDF contains information about the following supporting results, figures, analyses: **A**. Annotation Database Gene-Protein-Reaction (GPR) Agreement. **B**. Annotation Database Pathway Contribution. **C**. Experimental Data. **D**. Substrate Uptake Rates & Product Formation Rates. **F**. Flux Variability Analysis. Click here for file

Additional file 3 **Model SBML File**. This file contains the model in the Systems Biology Markup Language format. This file can be read into MATLAB using the COBRA Toolbox. Click here for file
