# Supplementary material for: Metabolic network reconstruction and genome-scale model of butanol-producing strain Clostridium beijerinckii NCIMB 8052
Source: BMC Syst Biol. 2011 Aug 16;5:130. doi: 10.1186/1752-0509-5-130 (PMC3212993; doi:10.1186/1752-0509-5-130)
Supplement: Additional file 2 — Additional Figures and Analyses. This PDF contains information about the following supporting results, figures, analyses: A. Annotation Database Gene-Protein-Reaction (GPR) Agreement. B. Annotation Database Pathway Contribution. C. Experimental Data. D. Substrate Uptake Rates & Product Formation Rates. F. Flux Variability Analysis. [file 1752-0509-5-130-S2.PDF]

Additional Data, Figures and Analyses

A: Annotation Database Gene-Protein-Reaction (GPR) Agreement

Figure A.1 shows the agreement for the gene-protein-reaction associations (GPRs) of each included reaction. One GPR is defined for each reaction, and a shared GPR is defined as an exact overlap (i.e., every associated gene is identical). **Figure A.2** shows the overlap for genes annoated in each database, irrespective of their associated reaction.

Figure A.1

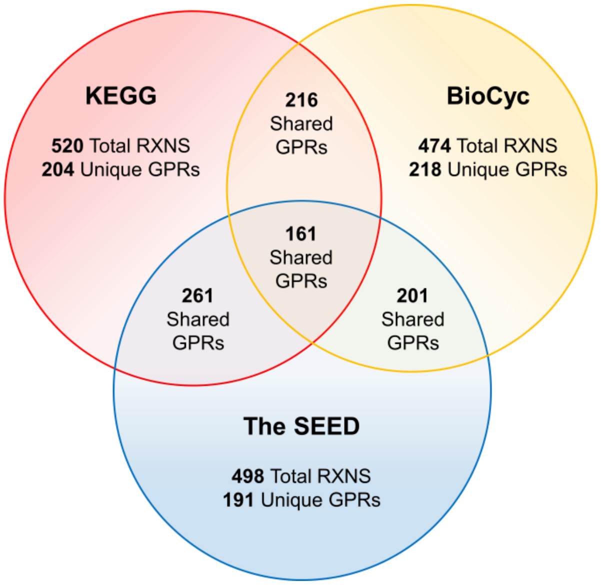

Figure A.2

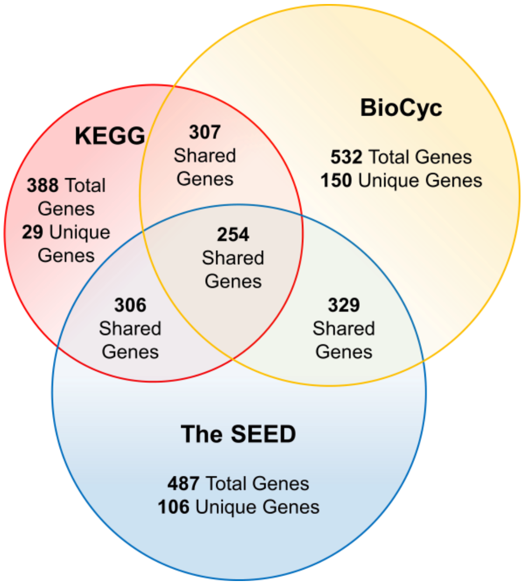

Additional Data, Figures and Analyses

B: Annotation Database Pathway Contribution

In addition to overall pathway counts, pathway counts for the the reaction “sets” from each annotation database were determined to investigate the contribution of each database in various areas of the network. The pathway distribution for each database was found to be similar, illustrating that each database performed similarly in all areas of metabolism.

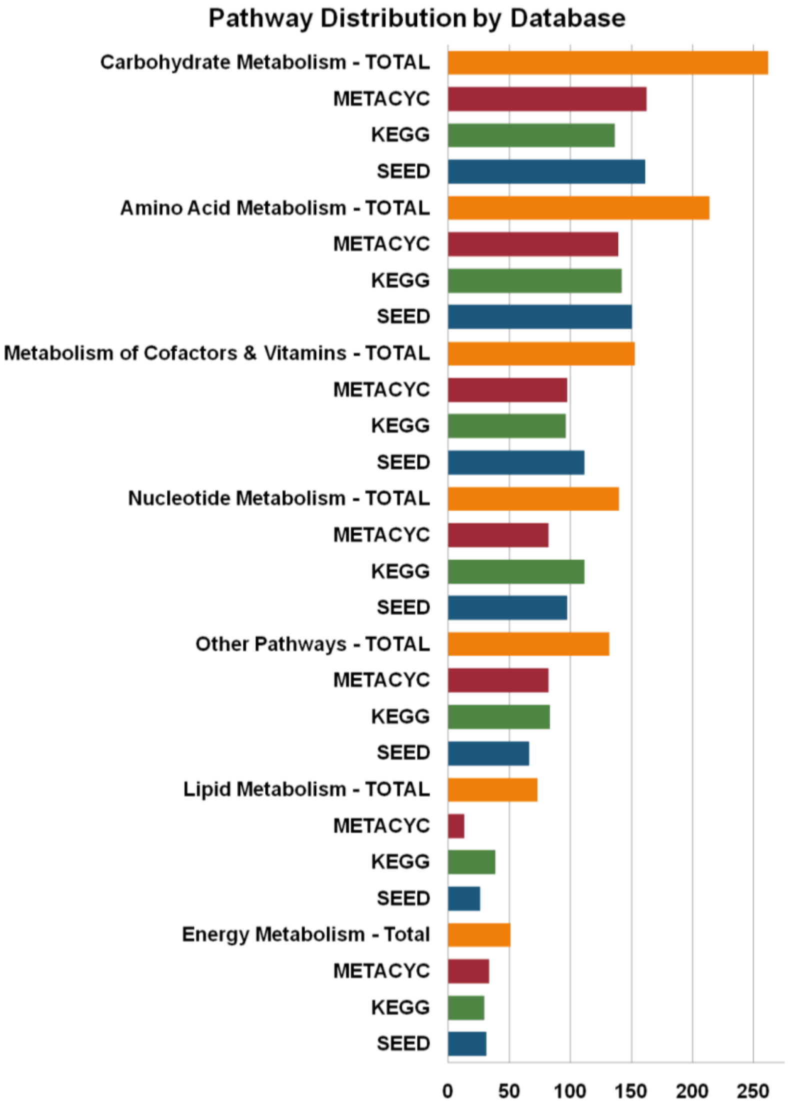

Additional Data, Figures and Analyses

C: Experimental Data

To obtain substrate uptake and product formation rates under multiple conditoins we We monitored concentration of glucose, acetate, butyrate, acetone, butanol, ethanol and biomass during batch fermentations at 30°C, 33°C, 35°C, and 40°C. See **Methods** for additional details. Presented data includes: growth curve and product concentration, glucose concetnration and pH profiles.

30°C Fermentation Profiles, I

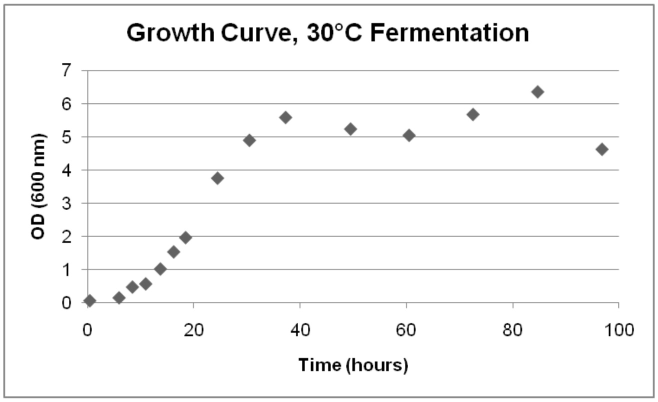

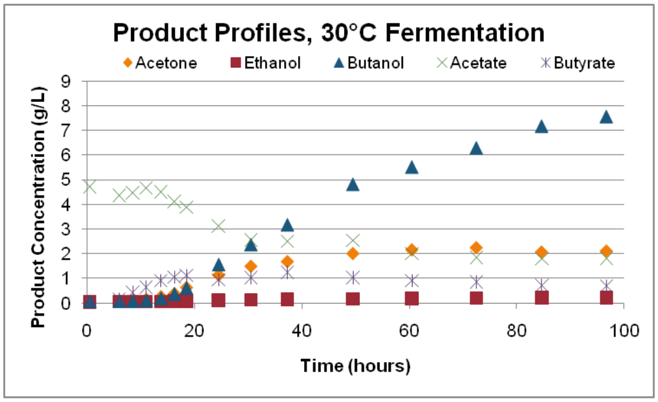

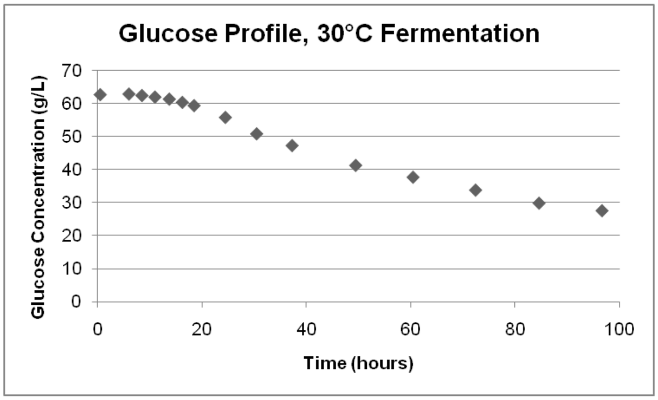

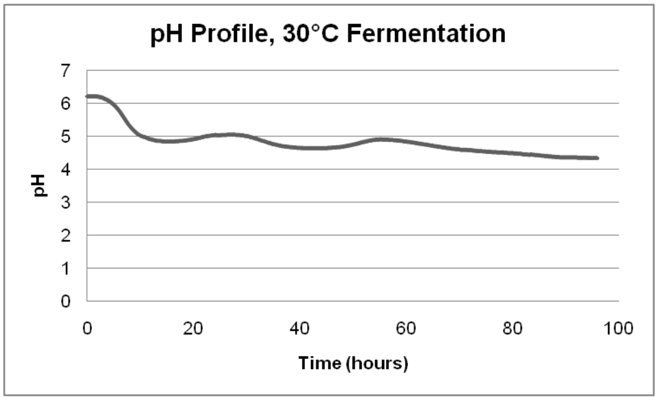

Additional Data, Figures and Analyses

30°C Fermentation Profiles, II

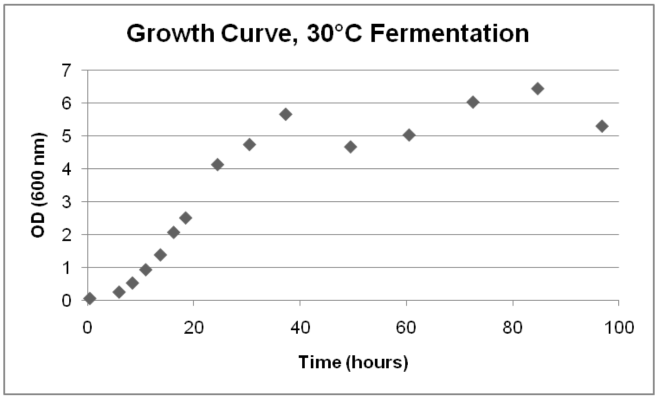

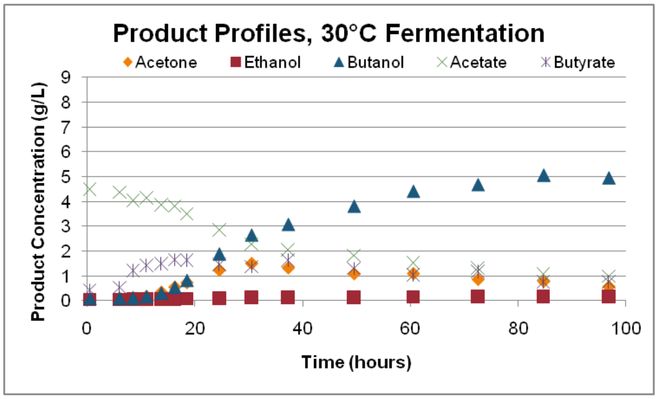

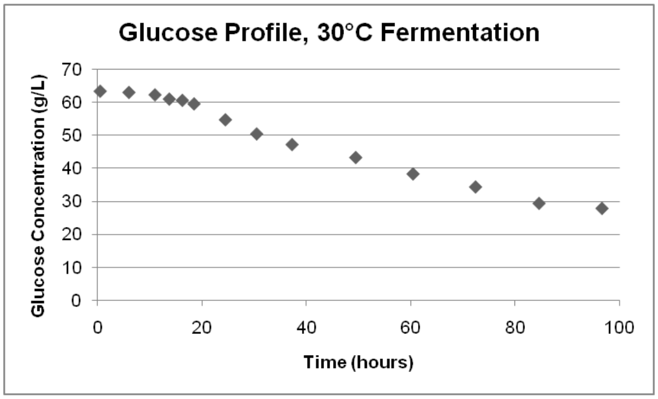

Additional Data, Figures and Analyses

30°C Fermentation Profiles, III

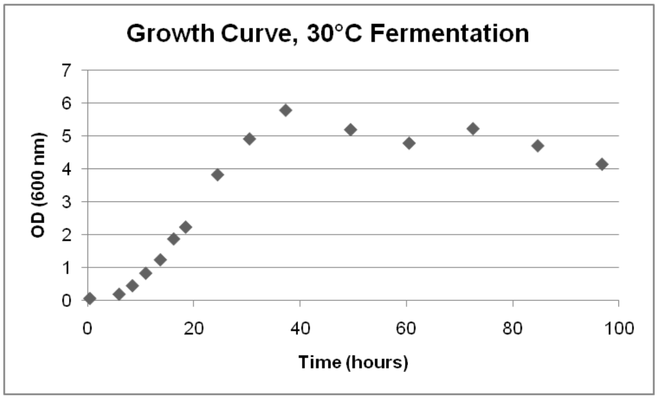

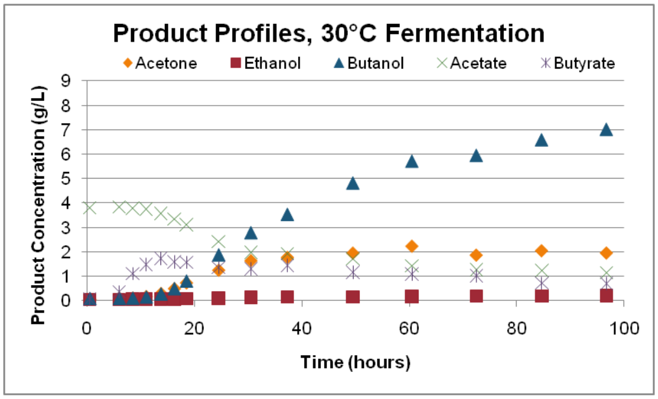

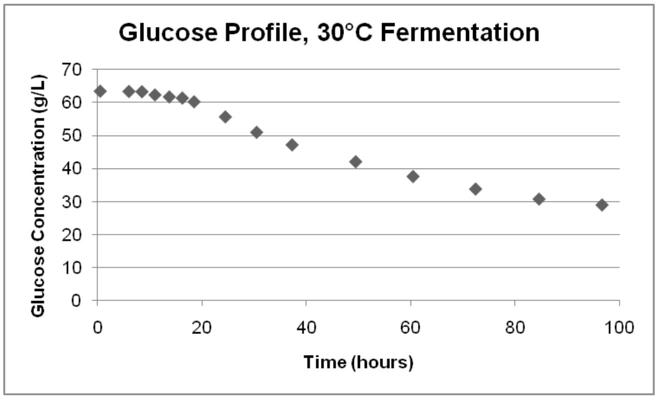

Additional Data, Figures and Analyses

33°C Fermentation Profiles, I

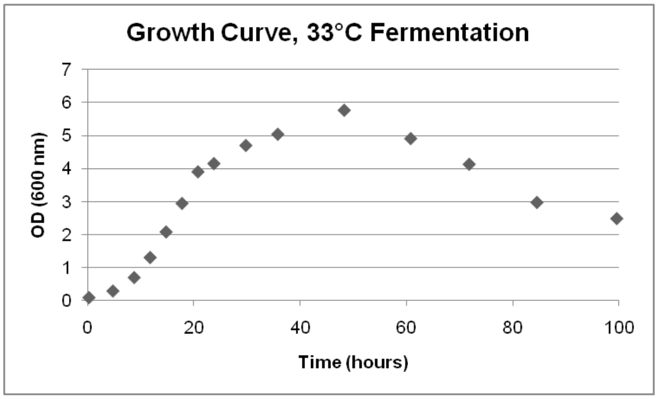

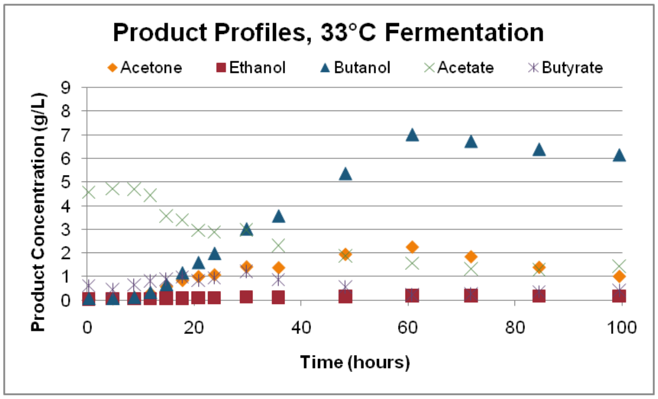

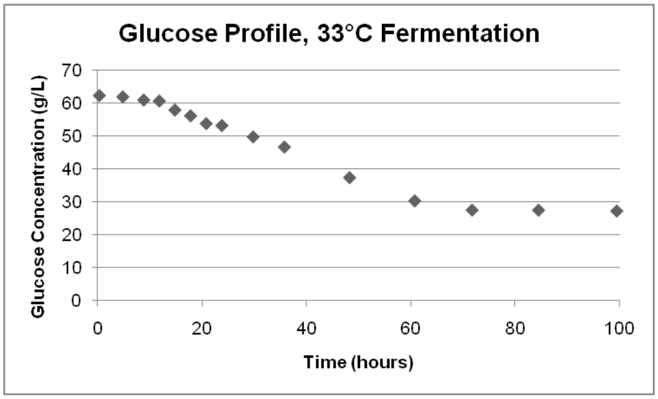

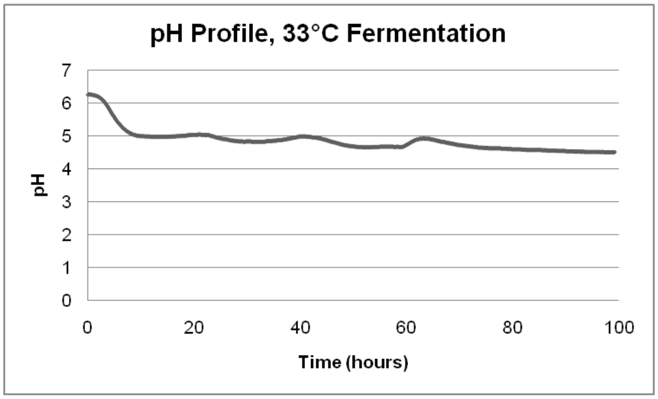

Additional Data, Figures and Analyses

33°C Fermentation Profiles, II

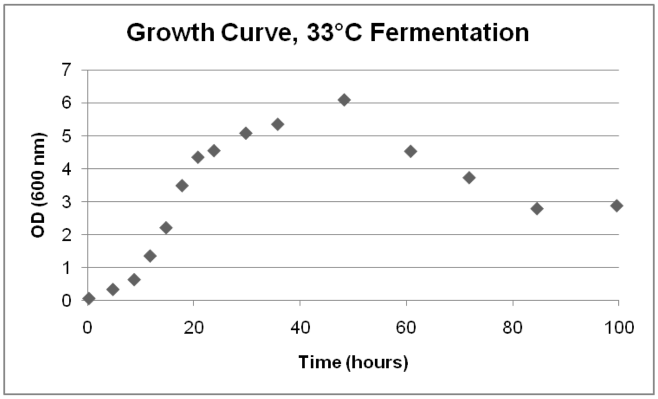

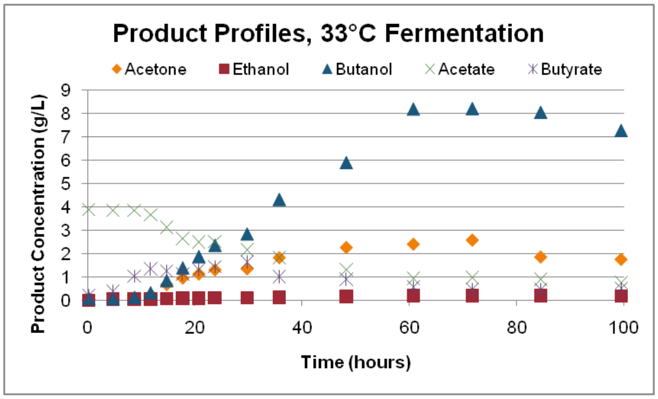

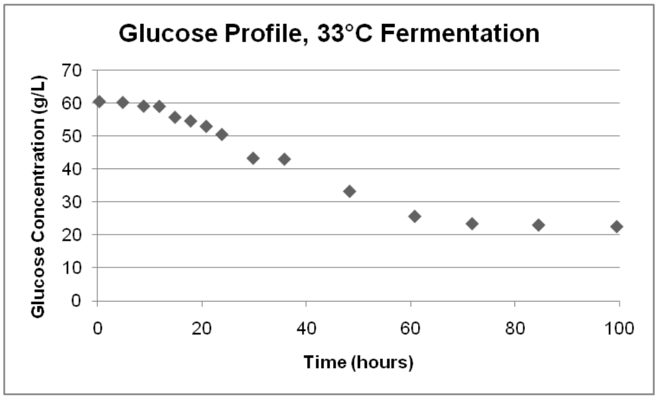

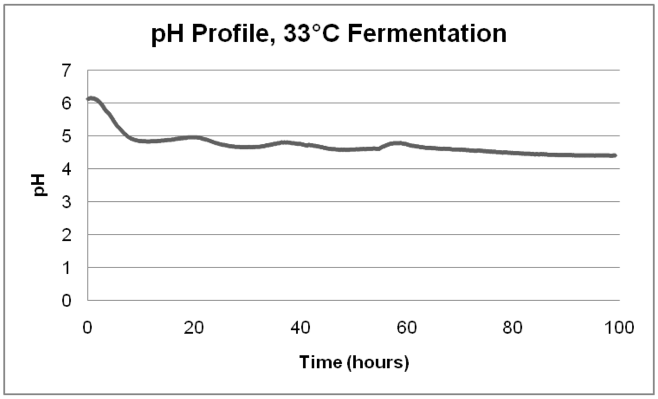

Additional Data, Figures and Analyses

33°C Fermentation Profiles, III

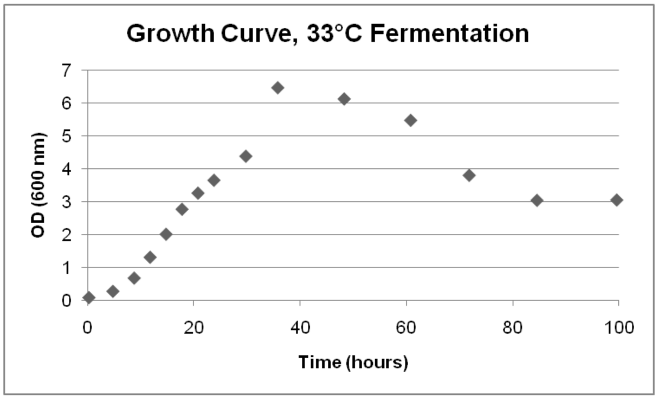

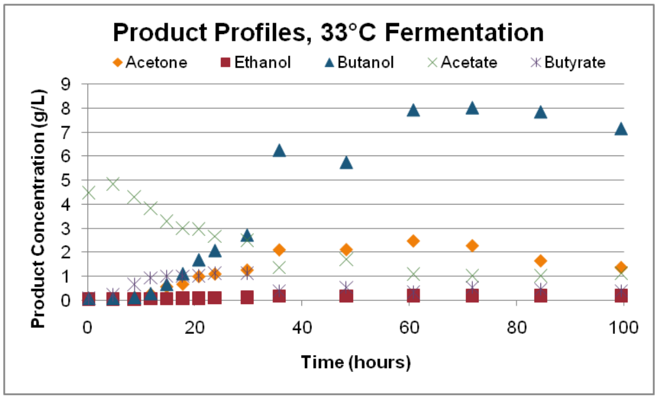

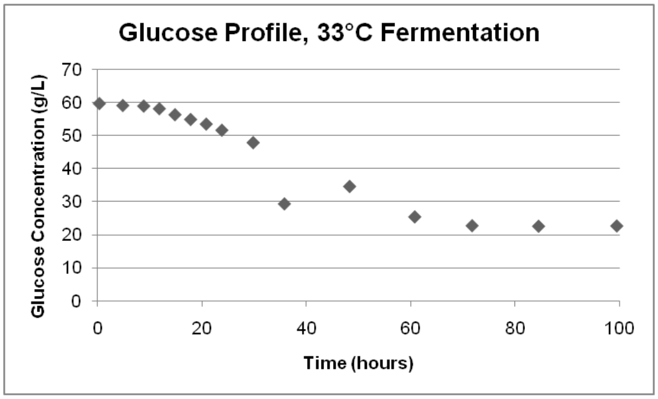

Additional Data, Figures and Analyses

35°C Fermentation Profiles, I

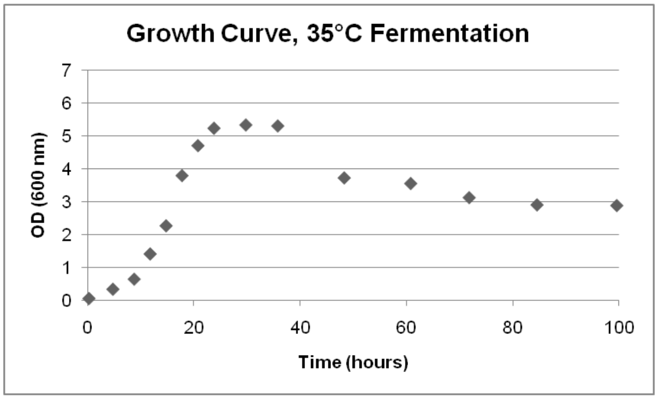

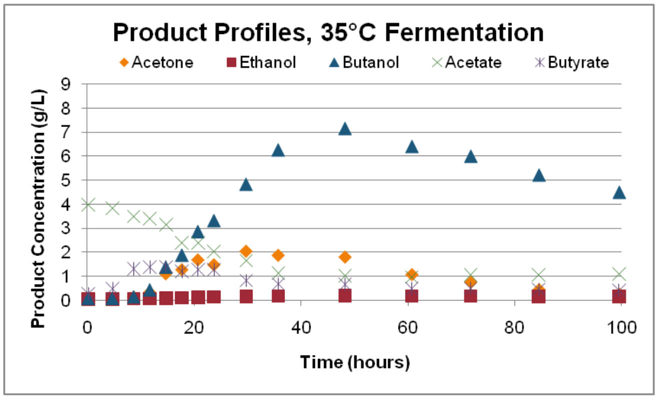

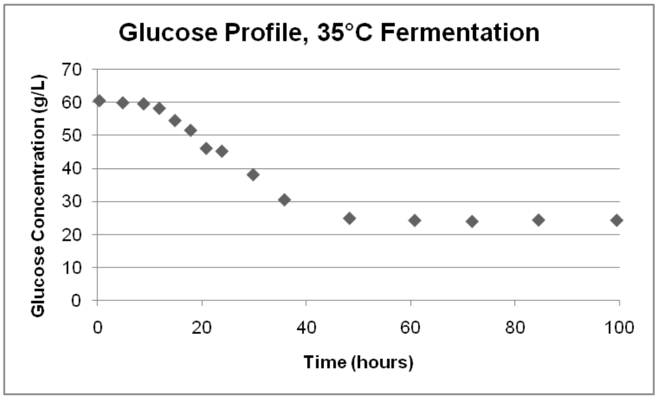

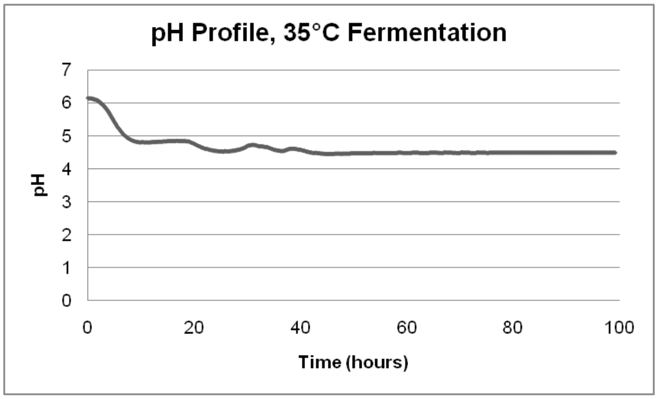

Additional Data, Figures and Analyses

35°C Fermentation Profiles, II

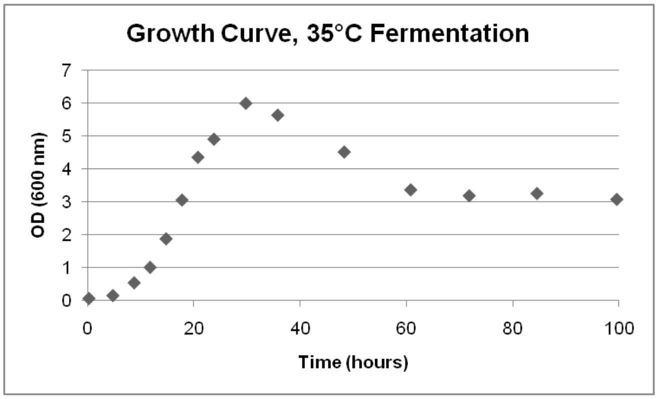

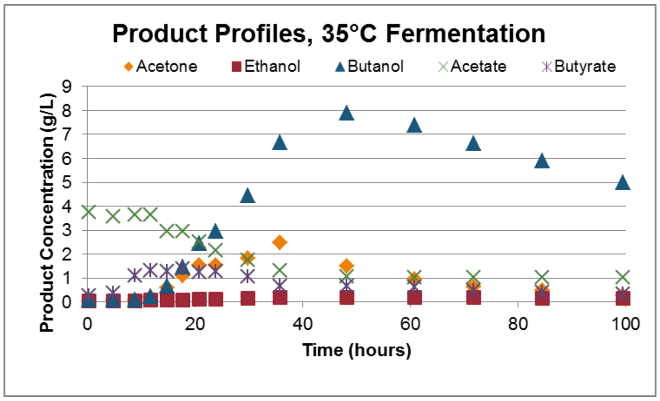

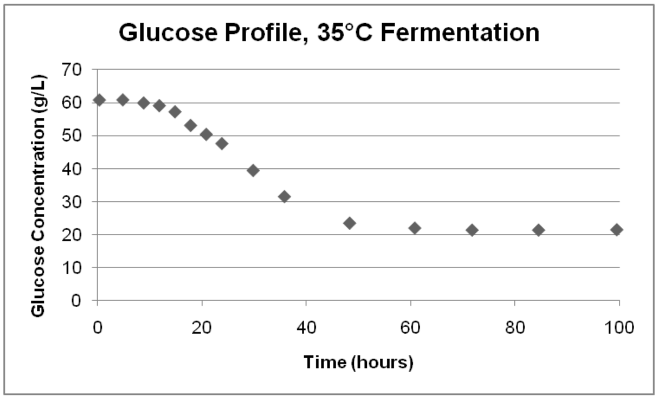

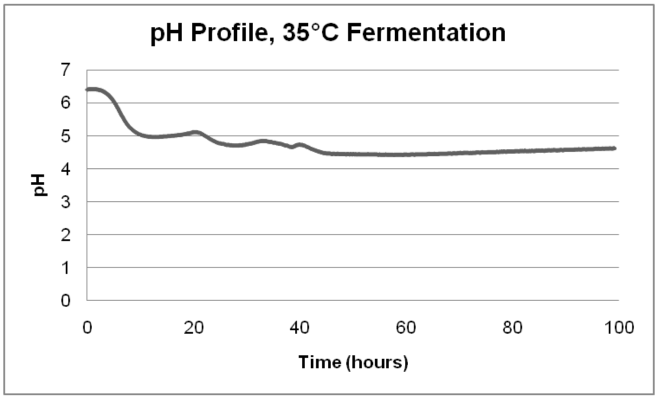

Additional Data, Figures and Analyses

40°C Fermentation Profiles, I

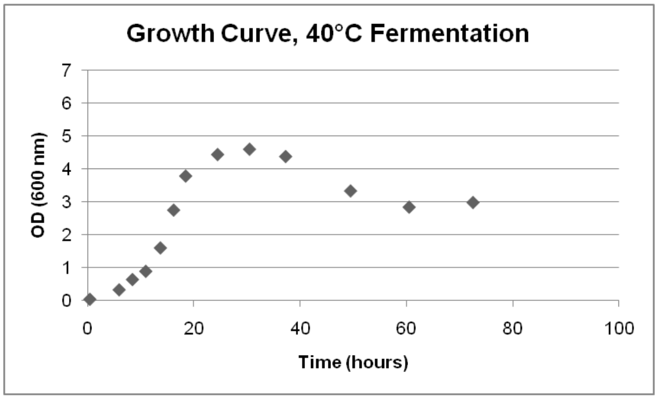

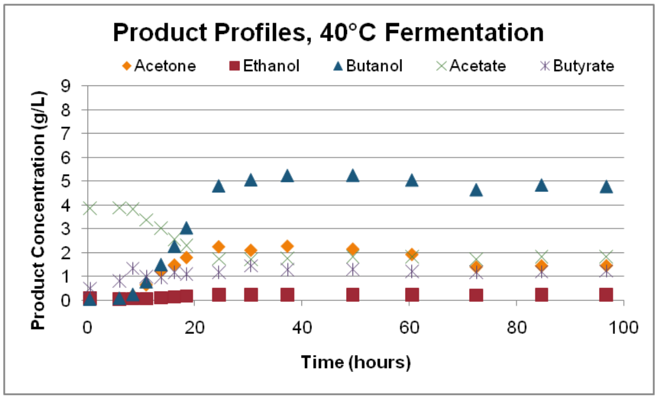

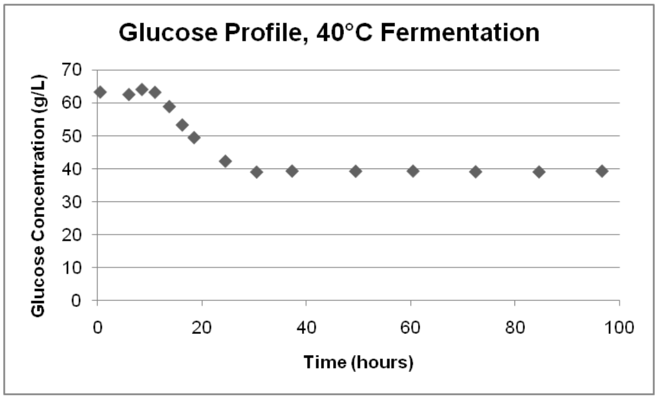

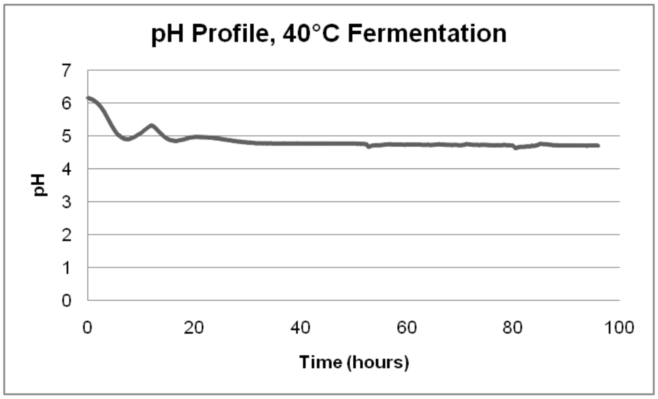

Additional Data, Figures and Analyses

40°C Fermentation Profiles, II

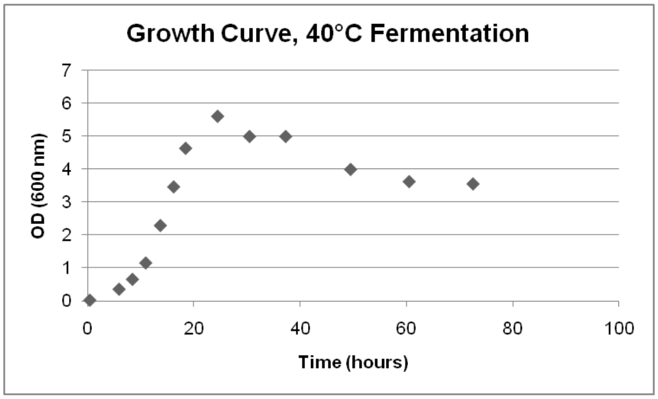

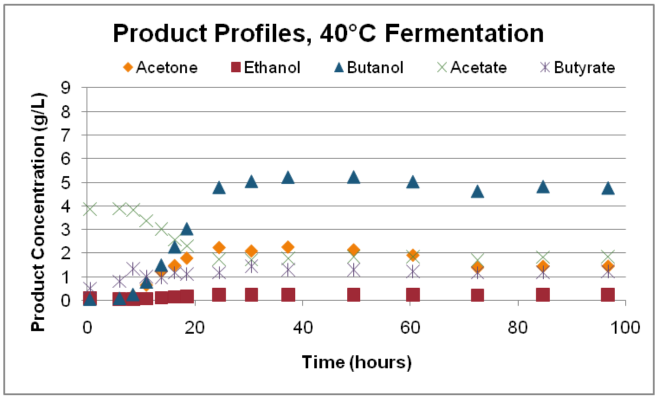

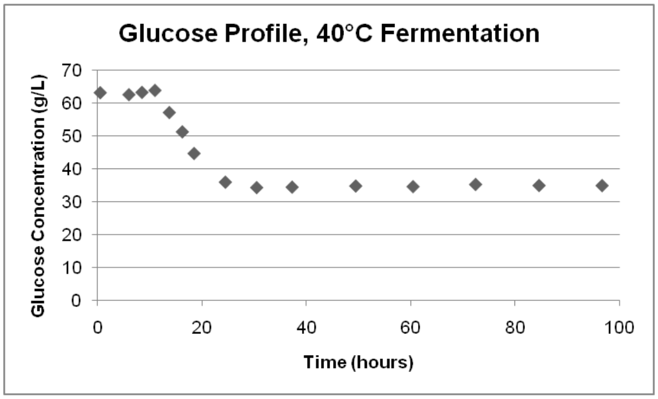

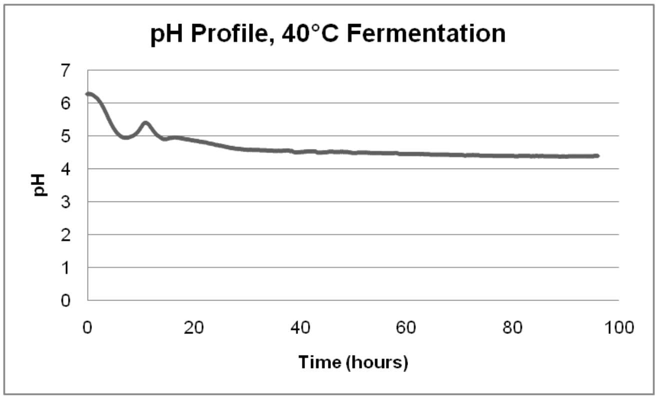

Additional Data, Figures and Analyses

40°C Fermentation Profiles, III

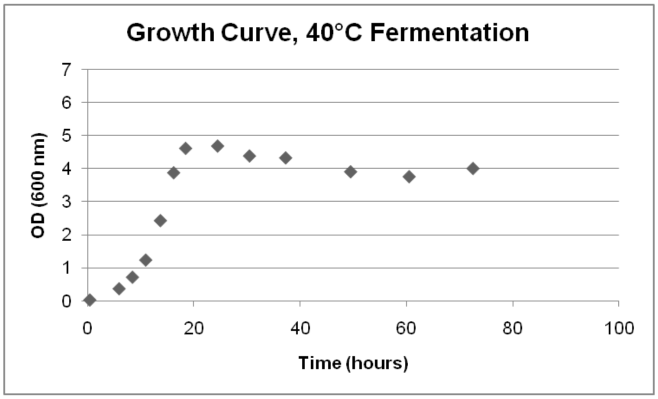

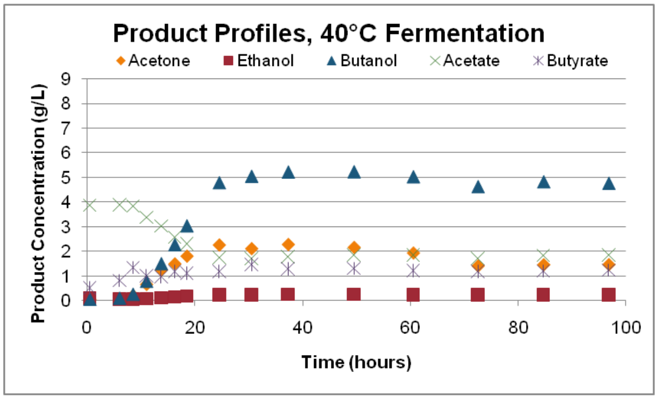

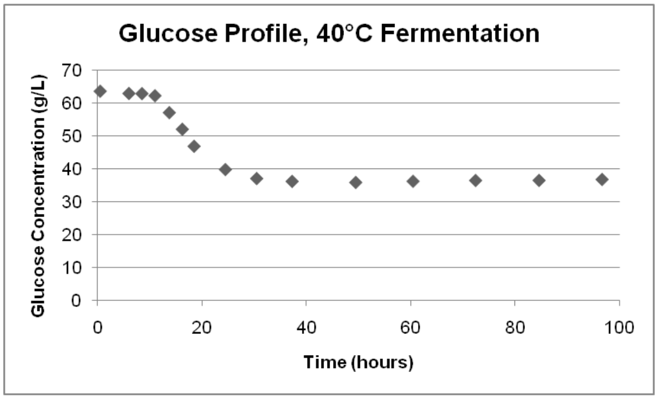

Additional Data, Figures and Analyses

D: Substrate Uptake Rates & Product Formation Rates

For each reactor we calculated the growth rate, glucose and acetate uptake rates, and acetone, ethanol, butanol and ethanol formation rates. These were calculated by multiplying yield and growth rate (see Methods), and averaged for each temperature. Inputs and outputs for the model were constrained to fall within one standard deviation of the average. Simulations run include all measured metabolites constrained with ATPM=0 and an ATPM value that aligns the simulated and experimental growth rates, as well as with only acetate and glucose constrained (ATPM=0). All rates are in units of mmol/gDW/hr.

		Growth	Glucose	Acetone	Ethanol	Butanol	Acetate	Butyrate
30 C	Reactor 1	0.127	-5.07	2.6	0.11	2.19	-2.56	2.35
	Reactor 2	0.105	-3.37	2.08	0.1	1.87	-1.8	0.95
	Reactor 3	0.106	-3.12	2.28	0.1	2.02	-2.59	0.96
	**Average**	**0.11**	**-3.85**	**2.32**	**0.10**	**2.03**	**-2.32**	**1.42**
	**Standard Deviation**	**0.01**	**1.06**	**0.26**	**0.01**	**0.16**	**0.45**	**0.81**
	Simulation Result, ATPM = 0	0.14	-4.91	2.06	0.09	1.87	-1.87	0.85
	Simulation Result, ATPM = 2.5	0.11	-4.91	2.06	0.09	1.87	-1.87	1.06
	Simulation Result, ac+glc only constrained, ATPM=0	0.17	-4.91	1.69	0	0	-1.87	2.97
		**Growth**	**Glucose**	**Acetone**	**Ethanol**	**Butanol**	**Acetate**	**Butyrate**
33 C	Reactor 1	0.116	-5.47	2.42	0.09	2.68	-4.58	0.69
	Reactor 2	0.148	-5.39	2.96	0.12	3.39	-4.06	0.00
	Reactor 3	0.105	-4.34	1.90	0.10	2.55	-4.20	0.69
	**Average**	**0.12**	**-5.07**	**2.43**	**0.10**	**2.87**	**-4.28**	**0.46**
	**Standard Deviation**	**0.02**	**0.63**	**0.53**	**0.02**	**0.45**	**0.27**	**0.40**
	Simulation Result, ATPM = 0	0.15	-5.7	2.96	0.12	2.77	-4.01	0.86
	Simulation Result, ATPM = 2	0.12	-5.7	2.96	0.12	2.96	-4.01	0.86
	Simulation Result, ac+glc only constrained, ATPM=0	0.18	-5.7	2.94	0	0	-4.01	3.47
		**Growth**	**Glucose**	**Acetone**	**Ethanol**	**Butanol**	**Acetate**	**Butyrate**
35 C	Reactor 1	0.16	-8.71	3.99	0.17	4.64	-3.61	0
	Reactor 2	0.165	-9.27	4.43	0.18	4.63	-3.44	0.68
	**Average**	**0.16**	**-8.99**	**4.21**	**0.18**	**4.64**	**-3.53**	**0.34**
	**Standard Deviation**	**0.00**	**0.40**	**0.31**	**0.01**	**0.01**	**0.12**	**0.48**
	Simulation Result, ATPM = 0	0.26	-9.39	3.90	0.17	4.63	-3.41	0.61
	Simulation Result, ATPM = 8.5	0.16	-9.39	4.42	0.17	4.63	-3.41	0.82
	Simulation Result, ac+glc only constrained, ATPM=0	0.32	-9.39	3.14	0.00	0.00	-3.41	5.68
		**Growth**	**Glucose**	**Acetone**	**Ethanol**	**Butanol**	**Acetate**	**Butyrate**
40 C	Reactor 1	0.106	-11.42	3.08	0.3	4.46	-3	0
	Reactor 2	0.164	-15.56	4.66	0.43	7.8	-4.45	-0.34
	Reactor 3	0.143	-10.44	3.47	0.34	5.79	-3.25	-0.21
	**Average**	**0.14**	**-12.47**	**3.74**	**0.36**	**6.02**	**-3.57**	**-0.18**
	**Standard Deviation**	**0.0294**	**2.71767**	**0.82306**	**0.06658**	**1.6815**	**0.77513**	**0.17156**
	Simulation Result, ATPM = 0	0.42	-15.19	4.56	0.43	7.7	-2.79	-0.01
	Simulation Result, ATPM = 20.5	0.14	-15.19	4.56	0.43	7.7	-2.79	-0.01
	Simulation Result, ac+glc only constrained, ATPM=0	0.53	-15.19	3.14	0	0	-2.79	9.64

Additional Data, Figures and Analyses

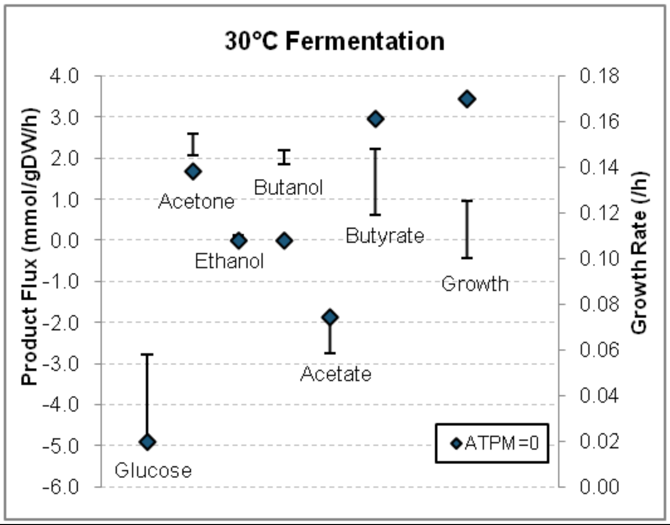

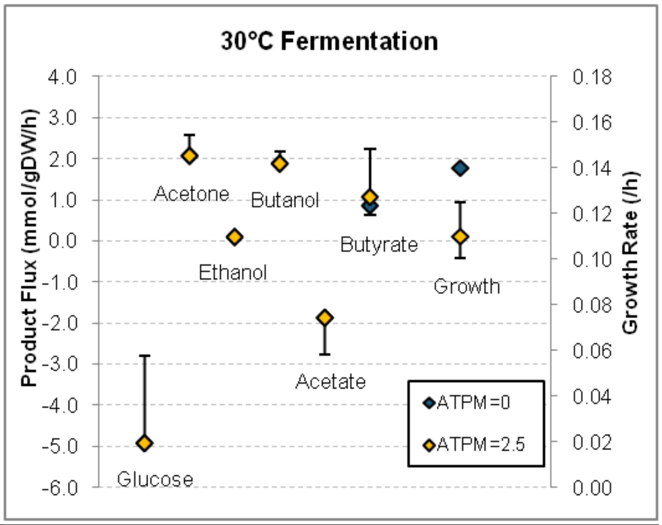

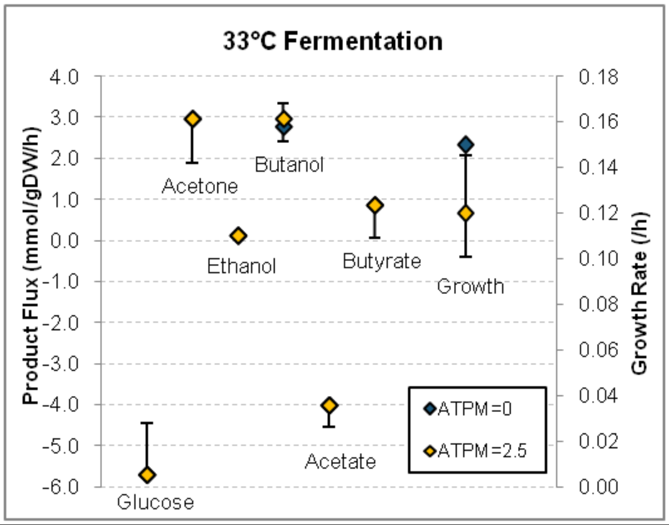

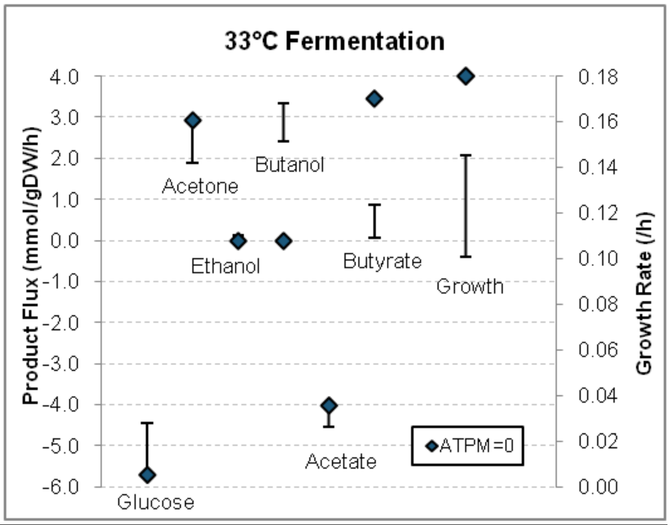

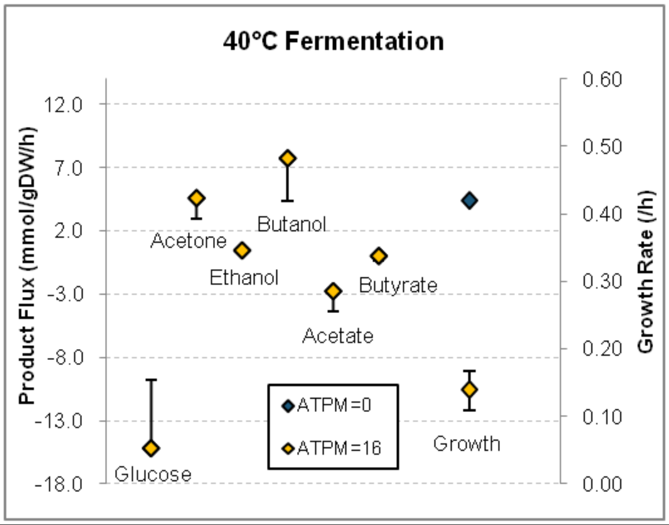

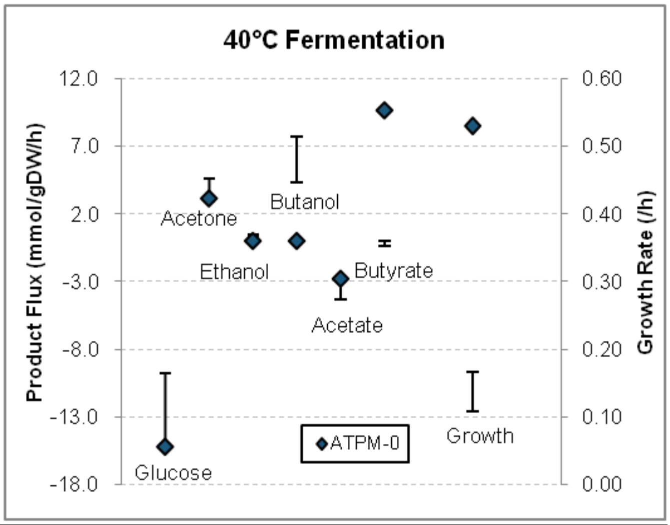

Additional Data, Figures and Analyses

E: Flux Variability Analysis

Flux Variability Analysis (FVA) was performed for the simulation where all measured inputs and outputs were constrained to the experimentally calculated values. Depicted below are reactions that could increase or decrease by 25% of their value predicted by FBA during optimal growth.

Reaction	Min	Max	Actual
ACCOAC	0	0.240469	0.24047
ACCOACDL	0	0.240469	0
ACGK	0.0176392	0.0414477	0.017639
ACHBS	0	0.195833	0.074017
ACK	-3.51612	1.00408	0
ACLS	0	0.195833	0.12181
ACOTA	-0.0414477	-0.0176392	-0.017639
AGPR	-0.0414477	-0.0176392	-0.017639
AHBNOOR	0	0.0740176	0
AHBPL	-1.22E-01	0.0740176	0
AHDHPT	-1.37E-32	0.002957	0
ALMM	0	0.121815	0
ALPL	-0.121815	0.0740176	0.00E+00
ASPO4	0	0.0037555	9.65E-32
ASPO5	0	0.0037555	0.0037555
BCOPBT	0.719299	5.23944	1.7234
BTNCC	0	0.240469	0
BUTK	0.719299	5.23944	1.7234
BUTOHDx	0	4.63007	4.63
BUTOHDy	0	4.63007	0
COAT1	0	4.52	3.516
COAT2	0	4.52	0.90337
DHDPRx	0	0.077931	0.077931
DHDPRy	0	0.077931	0
DHFR	0	7.61E-03	0.0076081
DHFRx	0	0.0076082	0
DHMBDH	-0.121815	2.12E-30	0.00E+00
DMPOR	-0.0740176	0	0
FAPNTPDH	0	0.002957	3.53E-33
FDH	0	16.7859	2.05E+00
FDHy	0	12.961	6.9053
FDXNRx	-19.7627	8.03942	3.50E-29
FDXNRy	0	12.961	0
FRD2	0	0.0038279	9.65E-32
FTHFL	0.0861169	0.125716	0.086117
G3PD1	0	0.241802	0
G3PD2	0	0.241802	0.15897
G3PD5	0	0.0828356	0
G5SD	0	0.0238004	0.0238
GALT	0	0.0425806	0
GALU	0	0.0425806	0.04258
GARFT	0	0.0395701	0
GARFT2	0.00E+00	0.0395701	0
GART	0	0.0395701	0.03957
GDH	-1.35E-32	0.002957	0
GLU5K	0	0.0238004	0.0238

Additional Data, Figures and Analyses

GLUSF	0	1.23296	1.2329
GLUSy	0	1.23296	0
GLYCLTDx	0	0.0828356	0.00E+00
GLYCTO2	0.002957	0.0857925	0.002957
GTP89H	0	0.002957	4.23E-33
GTPCI	0	0.002957	0.002957
HACD1x	0	5.45007	0
HACD1y	0	5.45007	5.45
HISTD	0	0.0082711	0.008271
HISTDOR	0	0.0082711	0
HISTOR	0	0.0082711	0
HSDx	0	0.263926	0
HSDy	0	0.263926	0.2639
Hex	0	0.0002171	0
KARA1	0	0.121815	0.12181
KARA2	0	0.0740176	0.074017
MTHFC	-0.0778749	-0.0382761	-0.038276
MTHFR2	0	0.0319094	0.031909
MTHFR3	0	0.0319094	0.00E+00
NADH16	0	0.0414178	0.041398
NDPK1	-8.62895	0.0656285	-1.63E-30
NDPK4	0.0046512	0.0245817	0.0046512
NDPK5	-8.6725	0.0220734	0
NDPK6	-0.0046994	0.0199305	0
NDPK7	0	0.0220734	0.0021429
NDPK8	-8.66999	0.0245817	0
NDPK9	0	0.0199305	0
NH42ex	0	0.0004342	0
ORNTA	0	0.0238004	0
ORNTAC	0.0175668	0.0414477	0.017639
P5CR	0	0.0238004	0.0238
P5CRx	0	0.0238004	0
PFL	0.109044	16.895	9.0615
PI2ex	0	0.0002171	0
POPT	0	8.67464	0.045698
POR4	0	16.7859	7.8334
PPNCL2	0	0.0017157	0
PPNCL3	0	0.0017157	0.0017157
PPND	0	0.0210997	0.0211
PPND2	0	0.0210997	0
PTA	-3.51612	1.00408	0
PYK	0	8.67464	8.6221
PYK2	0	8.67E+00	0.0046512
PYK3	0	8.67464	0.0021429
PYNP2	-0.0199064	7.24E-05	-0.019858
RNDR3	0	0.0022152	0.0021429
RNDR4	0	0.0046994	0.0046512
RNTR3	0	0.0022152	0
RNTR4	0	0.0046994	0
SHK3D	0	0.0493824	0.049373
SHK3Dx	0	0.0493824	0
TDPAT	0	0.195833	0
TRPS1	0	0.0033759	0.0033759
TRPS2	0	0.0033759	0

Additional Data, Figures and Analyses

TRPS3	0	0.0033759	0
UAPGR	0	0.0171629	0.017163
UAPGRx	0	0.0171629	0
UGLT	-0.0425806	0	0
UPPRT	0	0.0199305	0
URIK1	0	0.0199305	1.99E-02
URIK2	0	0.0199305	0
URIK3	0	0.0199305	1.33E-35
URIK4	0	0.0199305	0
URIK5	0	0.0199305	0
URIK6	0	0.0199305	0
URIK7	0	0.0199305	0
URIK8	0	0.0199305	0
URIK9	0	0.0199305	0
